# Ecotoxicological Effects of Four Commonly Used Organic Solvents on the Scleractinian Coral *Montipora digitata*

**DOI:** 10.3390/toxics11040367

**Published:** 2023-04-12

**Authors:** Valentina Di Mauro, Elham Kamyab, Matthias Y. Kellermann, Mareen Moeller, Samuel Nietzer, Laura H. Luetjens, Sascha Pawlowski, Mechtild Petersen-Thiery, Peter J. Schupp

**Affiliations:** 1 Environmental Biochemistry Group, Institute for Chemistry and Biology of the Marine Environment (ICBM), School of Mathematics and Science, Carl von Ossietzky University of Oldenburg, Schleusenstr. 1, 26382 Wilhelmshaven, Germany; 2 German Center for Marine Biodiversity Research (DZMB), Senckenberg am Meer, 26382 Wilhelmshaven, Germany; 3 Department of Product Safety, Regulatory Ecotoxicology, BASF SE, Carl-Bosch-Str. 38, 67056 Ludwigshafen am Rhein, Germany; 4 Product Stewardship and EHS Data Management, BASF Personal Care and Nutrition GmbH, Rheinpromenade 1, 40789 Monheim am Rhein, Germany; 5 Helmholtz Institute for Functional Marine Biodiversity (HIFMB), University of Oldenburg, Ammerländer Heerstr. 231, 26129 Oldenburg, Germany

**Keywords:** UV-filter, toxicity, biomarker, sunscreen, ecotoxicology, *Montipora digitata*, dimethylformamide, dimethyl sulfoxide, methanol, ethanol

## Abstract

Organic solvents are often used in aquatic toxicity tests to facilitate the testing of hydrophobic or poorly water-soluble substances such as ultraviolet (UV) filters, pesticides, or polycyclic aromatic hydrocarbons (PAHs). Knowledge of intrinsic effects (i.e., measured as standardized and non-standardized endpoints) of such carrier solvents in non-standardized organisms (i.e., corals), is critical to regulatory processes. Therefore, we exposed the reef-building coral *Montipora digitata* to the most commonly used carrier solvents ethanol, methanol, dimethyl sulfoxide, and dimethylformamide in the range of 10–100 µL L^−1^ for 16 days. The effects on mortality, photobiological, morphological, and oxidative stress markers were evaluated. In our study, all solvents resulted in significant morphological and/or oxidative stress responses, but not in mortality. Moreover, ethanol led to a rapid increase in turbidity, thus questioning its suitability as a carrier solvent in aquatic studies in general. Based on our observations, we could rank the solvent effects as follows: dimethylformamide < dimethyl sulfoxide ≈ methanol ≤ ethanol, with dimethylformamide showing the least and ethanol the most pronounced effects. We conclude that the use of solvents in toxicity studies with corals, particularly by examining non-standardized (e.g., morphological, physiological) endpoints, should be taken with caution and requires further elaboration.

## 1. Introduction

Coral reefs are considered as one of the most diverse and productive marine ecosystems, harboring at least one quarter of the marine biodiversity, despite covering only about 0.1% of the ocean surface [1,2]. The key to this astonishing diversity and productivity of coral reefs is largely attributed to the unique symbiosis of stony corals from the order Scleractinia with photosynthetically active dinoflagellates (i.e., zooxanthellae) [1,3].

Alarmingly, coral reefs have been threatened for several decades by anthropogenic stressors on both a global (e.g., rising sea surface temperatures) and local scale (e.g., overfishing, pollution) [3,4]. Stress-induced symbiotic dysfunction, such as the expulsion of vital symbionts (i.e., bleaching), inhibition of photosynthetic activities or even cell degradation, is a key phenomenon leading to the global decline of coral reefs [5,6,7]. Most recently, anthropogenic pollutants have attracted the attention of agencies and governments as a potential threat to coral reefs. Agricultural runoff, wastewater discharge, and coastal recreational activities are common sources of pesticides, pharmaceuticals, and personal care products with several containing hydrophobic or poorly water-soluble substances (e.g., ultraviolet (UV) filters, polycyclic aromatic hydrocarbons (PAHs)) [8,9]. Emitted into the aqueous phase, these compounds can easily be adsorbed by biological interfaces and various surfaces (e.g., glass) [8,9,10,11] and could therefore adversely affect coral reefs. However, in aquatic toxicity tests of hydrophobic compounds under both standardized and non-standardized test conditions, sometimes solvents are used to facilitate the substance delivery [10,12]. Most aquatic ecotoxicological studies have analyzed toxicity thresholds of several water-miscible organic solvents (e.g., ethanol (EtOH), methanol (MeOH), dimethyl sulfoxide (DMSO), dimethylformamide (DMF)) on aquatic standard organisms, such as fishes, crustaceans, or algae [13,14,15]. The outcome highlighted that solvent concentrations <100 µL L^−1^, as recommended by OECD [16], were not leading to adverse solvent effects within these surveys. If non-standardized test organisms, such as corals, are involved, these recommendations may differ. Thus, there are only few studies that address the health and survival of corals exposed to different solvents. In 2021, Machado and his colleagues [17] made initial efforts to establish thresholds for the safe use of solvents (i.e., MeOH, EtOH, and DMSO) in 96 h tests with tropical zoanthids, ranking EtOH as the most appropriate organic solvent for acute coral testing. Yet, it has not been studied how carrier solvents may affect reef-building (scleractinian) corals considering standardized (e.g., mortality) and non-standardized (e.g., physiology) endpoints. One of the health criteria for reef-building corals is their reliance on their algal symbionts [18]. Once exposed to stress, their symbionts downregulate metabolic processes as a protective response [19,20]. Depending on the severity of stress, it can lead to the disruption of the coral-algal symbiosis, which results in the loss of zooxanthellae in the host [21]. This phenomenon usually leads to a decrease in photosynthetic efficiency and a brightening of the host, widely known as coral bleaching. Therefore, visual morphological or photochemical examination of corals is critical and provides information about the health of the organisms as well as adverse effects such as coral pallor or tissue abnormalities [22,23,24]. Moreover, beyond pronounced adverse effects (e.g., bleaching, mortality), some authors suggested to include biomarker-based endpoints in laboratory tests with corals to gain more information on the coral’s inner physiology [25,26]. In conjunction with the use of carrier solvents, the recommended concentrations may lead to questionable results if the effect of the solvent as such on the test organism remains unclear [14]. Thus, the detection of early biochemical responses at different cellular levels makes it possible to determine the potential effects of stressors on the health status of the test organisms but may also lead to predicting the effects of stressors at higher levels [27,28,29].

Knowledge of basic toxicological conditions, such as the use of carrier solvents in testing hydrophobic substances, should be evaluated over a longer test period [16] with respect to the introduction of corals into regulatory processes. Therefore, this study focused on the effects of four commonly used solvents, namely EtOH, MeOH, DMSO, and DMF, on the scleractinian coral *Montipora digitata* during a prolonged exposure period of 16 days. Following the multidisciplinary approaches, we evaluated the effects of various targeted concentrations using standardized (i.e., mortality) and non-standardized (i.e., photobiological, morphological, and biochemical (i.e., oxidative stress responses) markers.

## 2. Materials and Methods

### 2.1. Husbandry and Supply of the Test Organism Montipora Digitata

Parental colonies of *M. digitata* stem from the long-term coral culture in the recirculating aquarium facilities of the Environmental Biochemistry working group at the Institute for Chemistry and Biology of the Marine Environment (ICBM) (University of Oldenburg, Wilhelmshaven, Germany). The aquarium facilities were run with reconstituted artificial seawater using Tropic Marin^®^ Pro-Reef salt (Wartenberg, Germany). In order to maintain a stable water chemistry, element and micronutrient dosing was performed via Balling system with programmable peristaltic pumps (Reef Doser Evo 4, Aqua Medic GmbH, Bissendorf, Germany). Water quality parameters (see Table S1, Supplementary Material Section S1.1) were closely monitored via in-house testing and externally with inductively coupled plasma optical emission spectrometry (ICP-OES) analyses (Prof. Dr. Biener GmbH, Leubsdorf, Germany). LED panels were used for illumination (Radion G4Pro, EcoTech Marine LLC, Bethlehem, PA, USA) to provide a light intensity reaching a photosynthetic photon flux density (PPFD) of 100–130 ${µ\mathrm{mol}\;s}^{-1}{\;m}^{-2}$. The water temperature was kept at 26 ± 1 °C and the salinity was 34.5 ± 1 practical salinity unit (psu). *M. digitata* is a branching species but can be grown encrusting as well. To prepare coral fragments for the experiment, mother colonies were grown encrusting on PVC plates. The PVC plates were kept in the general aquarium facility. Coral fragments were produced using a hollow drill of the desired diameters (ø 14–16 mm). The obtained coral fragments were fixated on glass plates (300 cm^2^) using cyanoacrylate glue (Spezial 483-HV, 2construct GmbH, Germany) followed by a minimum 7–14 day acclimation and recovery period under experimental conditions. The experimental conditions closely followed the culture conditions of the mother colonies and adhered to the husbandry recommendations described in Table S2 (Supplementary Material Section S1.2).

### 2.2. Experimental Set Up

For organizational reasons, the experiments were conducted sequentially. The set of experiments were performed with EtOH, MeOH, and DMSO, while the second used DMF. A control group was included in both sets to allow for a cross comparison of the data (i.e., reproducibility of control results).

#### 2.2.1. Preparation of Test Media and Controls

**Figure 1:**
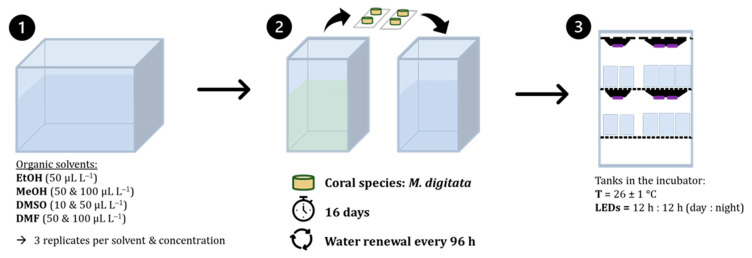
Schematic overview of the long-term experiment described here using *Montipora digitata* as the test organism. Coral discs (14–16 mm in diameter) were fixated on a glass slide and placed in a glass tank containing 1 L of water. The experiment was conducted over a period of 16 days, with the seawater replaced every 4 days (96 h).

In this study, we tested the commonly used carrier solvents, such as methanol (MeOH, Biosolve Chimie SARL, 99.98% in ULC/MS-CC/SFC grade, Dieuze, France), ethanol (EtOH, Carl ROTH GmbH & Co. KG as ROTIPURAN^®^, 99.8%, Karlsruhe, Germany), dimethyl sulfoxide (DMSO, Carl ROTH GmbH & Co. KG as BioScience-Grade, 99.5%, Karlsruhe, Germany), and dimethylformamide (DMF, VWR International S.A.S., 99% in HPLC grade, Rosny-sous-Bois, France). The following targeted solvent concentrations were prepared: 50 µL L^−1^ (0.005% *v*/*v*) of all solvents, 100 µL L^−1^ (0.01% *v*/*v*) of MeOH and DMF, and 10 µL L^−1^ (0.001% *v*/*v*) of DMSO dissolved in filtered artificial seawater (FSW; filter disk diam. 0.47 mm NY 0.22 µm, Nylon (NY) Membrane, GVS, Zona Industriale, Italy) from the mother colony tanks of the aquarium facility (Figure 1, No.1). The chosen test concentrations were either closely related to the recommended solvent concentration of 100 µL L^−1^ [16] when data were not available (i.e., for DMF) or to previous performed aquatic toxicity tests using carrier solvents, such as MeOH [30] or DMSO [31]. The concentration for EtOH was based on inhouse preliminary tests that showed a proportional increase in turbidity with increasing concentration. Only moderately increased turbidity during the 96 h test was evident at a concentration of 50 µL L^−1^.

#### 2.2.2. Static Renewal System

After successful acclimation and recovery, four randomly selected *M. digitata* discs (two microscope slides with two fragments each) were placed in each replicate (three replicates per treatment (i.e., MeOH, EtOH, DMSO, and DMF) and in each of the six control replicates (i.e., negative). A total number of 27 tanks were used. During the 16 day exposure, coral fragments were kept under a static-renewal system, which allowed for the renewal of the seawater in each tank completely every 96 h (Figure 1, No. 2). In order to maintain the same environmental conditions of temperature (at 26 ± 1 °C), oxygen content (>80% saturation), and light intensity (PPFD of 100–130 ${µ\mathrm{mol}\;s}^{-1}{\;m}^{-2}$) as in the aquarium facility, the experiment was conducted in an incubator (Panasonic MIR-554-PE, PHC Europe B.V., Etten-Leur, The Netherlands) (Figure 1, No. 3). Water circulation and aeration was provided by an air tubing system connected to a lubricant-free air compressor (MEDO LA-45B, Nitto Kohki Co., LTD, Ohta-ku, Tokyo). A 12 h light:dark period, including a sunrise and sunset phase of 45 min, was set with the same type of LED (Radion G4 Pro, xr15 mono panel, xr30 double panels; EcoTech Marine LLC, Bethlehem, PA, USA) as in the aquarium facility, which emitted a photosynthetic photon flux density (PPFD) of 100–130 ${µ\mathrm{mol}\;s}^{-1}{\;m}^{-2}$ on the coral surface level. Light intensity and temperature were monitored using the HOBO pendant data logger (see Supplemental Material Section S.1.3.).

### 2.3. Water Quality

#### 2.3.1. Water Parameters

To capture confounding factors associated with the tested solvents, we monitored water quality at the beginning and end of the 96 h renewal period (in 16 days of exposure) using the following water parameters: dissolved oxygen (DO), pH, salinity (Sal), alkalinity (ALK), calcium (Ca), and nutrients, i.e., phosphate ($PO_{4}^{3-}$) and nitrate ($NO_{3}^{-}$). DO, pH, and Sal were measured using a WTW MULTI 3630 IDS device connected to an FDO 925-P probe, a SensorLyt 900-P probe, and a TertraCon 925-P probe. Nitrate was determined by measuring the concentration of $NO_{3}^{-}$ (+$NO_{2}^{-}$) by adapting the assay from Schnetger and Lehners [32]. ALK and Ca were analyzed using titration SALIFERT^®^ Profi Test Kits. HANNA INSTRUMENTS^®^ HI736 Phosphorous Measurement in ultra-low range (0–200 ppb) were used for measuring $PO_{4}^{3-}$ concentrations.

#### 2.3.2. Turbidity

Previous studies have shown that some solvents can serve as additional carbon sources and promote the growth of microorganisms such as bacteria or algae [14]. In addition, the increased growth of microorganisms can lead to a deterioration in water quality, corelating with increased turbidity [33]. This parameter was measured by optical density (OD) at a 600 nm wavelength using a microplate reader (Synergy H1 from BioTec instruments, Santa Clara, CA, USA). For purpose, 200 µL samples from each treatment and control were taken from the center of the tank after 0, 6, 18, 24, 48, 72 and 96 h, respectively, and transferred into the cavities of a 96 well-plate.

### 2.4. Coral Health Indicators as Biological Responses at the Organismal and Cellular Levels

To assess the coral health status on the organismal and cellular level, we followed multi-disciplinary approaches using photobiological, morphological, and also biochemical (i.e., oxidative stress responses) markers.

#### 2.4.1. Organismal Level

##### Photosynthetic Efficiency of Symbiotic Algae/Symbionts

The photobiological response on the organismal level was estimated through fluorometric measurement using pulse amplitude modulated (PAM) fluorometry (MINI-PAM; Heinz Walz GmbH, Effeltrich, Germany). At the beginning and at the end of each 96 h renewal phase, photosynthesis activity was measured as a non-intrusive method. Here, the effective photochemical quantum yield ($\Phi_{PSII}$) was determined as an indicator of the actual efficiency of the electron transport system in photosystem II (PSII) of the symbionts zooxanthellae while using the fiberoptic with an active area of 7 mm [34]. During the measurement, the fiberoptic was held 5–8 mm above the coral fragment submerged in the water, while avoiding touching or scratching the coral surface. For each fragment, five values were collected by scanning different areas of the coral surface. The $\Phi_{PSII}$ was calculated with $F_{M}^{\prime }$ as the maximum fluorescence level of the PSII reaction centers during saturation pulses and F as the momentary fluorescence level shortly before the saturation pulse, as described in the study performed by Matyssek and colleagues [35]:

(1) $$\Phi_{PSII}=\frac{F_{M}^{\prime }-F}{F_{M}^{\prime }}$$

For comparing photobiological responses through PAM fluorometry between 2 datasets (here, 2 experiments), the values were aggregated and transferred into 0–100% range. This was performed by transforming the values in a Min-Max scale within each experiment, using the simple formula:

(2) $$\%(\Phi_{PSII})=\left(\frac{x-\min\Phi_{PSII}}{\max\Phi_{PSII}-\min\Phi_{PSII}}\right)*100$$

with, x = measured values, $\min\Phi_{PSII}$= smallest, and $\max\Phi_{PSII}$= largest measured value within each data set.

##### Morphological Analysis

A non-intrusive morphological study was conducted at the beginning and at the end of the experiment by photo analyses of fragments and polyps using a digital camera (Olympus TG-5, Olympus Europa SE & Co. KG, Hamburg, Germany) and a digital microscope camera (200X 8-LED microscope endoscope MS100 Camera, Shenzhen, China) with 200x magnification, respectively. The fragment morphology was studied based on three visual aspects: paleness, surface polyp density (including polyp losses), tissue contraction/alterations, and report of mortality. A total number of 12 fragments per solvent concentration and 24 fragments of control were analyzed. Abnormalities in polyp morphology were studied from the following two angles: pigmentation (e.g., homogeneity) [3,36,37] and the structures of their tentacles. All observations were reported and related to the control group.

#### 2.4.2. Cellular Level

##### Sample Preparation for Biomarker Analyses

Adopting the protocols and procedures described for different marine organisms [27,38,39], we measured the oxidative stress responses (i.e., lipid peroxidation (LPO), catalase (CAT)), and the overall cellular energy allocation (CEA) as indicators for coral health status. Exposed fragments were transferred to 24-well plates sealed with parafilm and immediately frozen and stored at −80 °C until further analysis. Before proceeding with any bioassays, each fragment obtained was weighted and kept on ice for the following homogenization process. A total of 1.5 mL PBS buffer (0.137 M NaCl, 0.0027 M KCl, 0.01 M Na_2_HPO_4_, 0.0018 M KH_2_PO_4_ in ultra-pure water, pH = 7.4) was added stepwise while using a grinder (Roti-Speed-stirrer, 5000–20,000/min, Carl Roth GmbH, Karlsruhe, Germany) for the homogenization of the entire fragment, including both the tissue and skeleton. During homogenization, the samples were kept cold using a frozen metal rack. Following Dias and colleagues [38], we centrifuged the homogenized samples by 10,000× *g* relative centrifugal force (RCF; g-force) at 4 °C for 15 min. From the receiving supernatant, aliquots were separately collected as followed:

- 150 µL for LPO, in which 2.5 µL butylated hydroxytoluene (BHT) was added.
- 170 µL for CAT, including 30 µL for total protein (Pr) quantification.
- 450 µL for CEA divided into:
  ○ 150 µL for electron transport system (ETS) activity.
  ○ 150 µL for carbohydrate and protein (CH/Pr) quantification.
  ○ 150 µL for lipid (LIP) quantification.

##### Oxidative Stress Responses (LPO; CAT)

As an indicator of oxidative damages, LPO levels were measured. Adapting the methods from Ohkawa, et al. [40], Bird [41], and Torres, et al. [42], LPO was obtained by measuring thiobarbituric acid (TBA) reaction substances (TBARS) at 535 nm, which was produced by the lipid oxidation product malonaldehyde (MA) reacting with the TBA 0.73% reagent. The results were calculated using a molar extinction coefficient of 1.56 × 10^5^ M/cm and expressed as nmol TBARS/mg of frozen weight (fw) coral fragment.

Quantifying the activity of CAT was based on the method described in the study of Claiborne [43]. Here, CAT activity was measured by detecting the degradation of H_2_O_2_ during 3 min at 240 nm. The protein content of each sample was measured according to Bradford [44] adapted to the Bio-Rad micro assay using bovine serum albumin (BSA) as the standard solution by 600 nm. Finally, CAT activities were expressed in μmol/min/mg of protein, using a molar extinction coefficient of 40 M cm^−1^.

##### Cellular Energy Allocation (CEA)

The CEA method was applied to detect the health status of coral fragments under in-vitro incubation conditions. CEA represents the ratio between the energy reserves availability E_a_ (i.e., CH, Pr and LIP), and the energy consumption E_c_ (i.e., ETS), within an organism. The procedures for the measurement were adopted and optimized from DeCoen and Janssen [45].

Energy availability (*E_a_*):

CH and protein were determined from the same aliquoted sample, as the sample was pre-treated using 15% tetrachloroacetic acid (TCA) for receiving a CH and protein fraction. The content of CH was determined by adding 5% phenol and concentrated H_2_SO_4_ (99.95%) to the CH fraction, measured at an absorbance of 492 nm while using α-D-Glucose as a standard solution. The protein content was determined in the protein fraction while treated with 1.67 M hydrochloric acid (HCl) and 1 M sodium hydroxide (NaOH) at 592 nm using BSA as a standard solution, which was modified from the Bradford [44] method. The total LIP content was measured at 375 nm according to a modified bioassay described initially by DeCoen and Janssen [45] using a chloroform-based extraction method with tripalmitin as standard. A microplate reader (Synergy H1 from BioTec instruments, Santa Clara, CA, USA) was used for measuring each molecular marker.

Energy consumption (*E_c_*):

Following a modified bioassay from DeCoen and Janssen [45], ETS was measured by adding NADPH and iodonitrotetrazolium (INT) at room temperature as a reactant to the sample at 490 nm for 3 min. Here, the formation of 2 µmol formazan is equivalent to the consumption of 1 µmol O_2_ in this reaction. Subsequently, we converted the obtained oxygen consumption rate into caloric values using oxy-enthalpic equivalents of 484 kJ/mol O_2_, which accounts for an average carbohydrate, lipid, and protein mixture [46].

Ultimately, the relationship between available energy *E_a_* and the rate of energy consumption *E_c_* was integrated into the cellular energy allocation value, using the following equation:

(3) $$\mathrm{CEA}=\frac{E_{a}}{E_{c}}\;\left(\mathrm{mJ}/\mathrm{mg}\;\mathrm{of}\;\mathrm{fw}\;\mathrm{coral}\;\mathrm{fragment}\right)$$

### 2.5. Statistical Analyses

Normal distribution and homogeneity of the variances were tested using a Shapiro test followed by a Bartlett test (normal) or Levene test (non-normal), depending on data distribution. ANOVA (homogeneous) or Welch ANOVA (heterogenic) were used for analyzing the statistical significances of data sets. Statistical differences for treatments (i.e., solvent doses) against controls and within treatments were analyzed using a TukeyHSD (homogeneous) or GamesHorwell (heterogenic) post-hoc test. When data showed a non-normal distribution, significances were estimated by using the Kruskal–Wallis test followed by Dunn´s post-hoc test. The significance was set to ≤0.05 while pairwise comparisons were adjusted to the “BH” method, when necessary. The statistical results were summarized in the Supplemental Material Section S.3.

Statistical analyses were performed by RStudio (R software version 4.2.2, PBC, 31.10.2022) using R packages Performance [47], PMCMR [48], conover.test [49], dunn.test [50], and ggplot2 [51].

## 3. Results

No mortality was observed in any of the controls or treatments within the 16 days test duration.

### 3.1. Water Quality

#### 3.1.1. Water Parameters

The general water chemistry was maintained within the acceptable limits of water quality throughout the experiment, as recommended by Borneman [52] and presented in the supplementary material S.1.2. However, the following water quality parameters, namely Sal, O_2_, pH, Ca, and ALK remained >80% of initial concentrations within each 96 h water renewal period (see Supplementary Material Section S.1.4, Table S4). In contrast, nutrient concentrations (i.e., $PO_{4}^{-3}$, $NO_{3}^{-}$) declined by more than 70% after 96 h. The reduction in $NO_{3}^{-}$ was in fact higher at 50 µL L^−1^ EtOH, while $PO_{4}^{-3}$ depletion was strongest in 50 µL L^−1^ of DMF, compared to other treatments (i.e., control, solvents).

#### 3.1.2. Water Turbidity via OD Measurement

**Figure 2:**
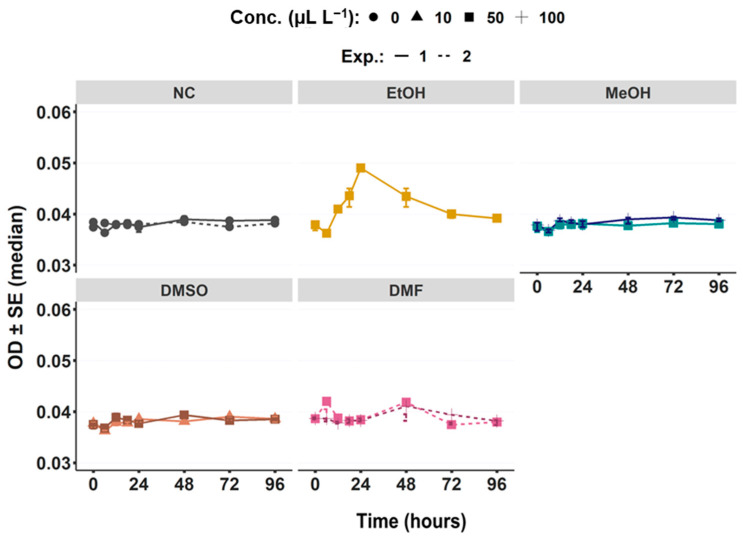
Measured water turbidity (i.e., optical density (OD), at 600 nm) within 96 h renewal intervals for the negative control (NC) and the solvent experiments (i.e., ethanol (EtOH), methanol (MeOH), dimethyl sulfoxide (DMSO), and dimethyl formamide (DMF)).

The OD data were non-normal distributed (W = 0.511; *p* ˂ 0.05) with no homogeneity (F = 1.49; *p* ˂ 0.05) in variances and therefore displayed as median values. Significant differences were found regarding treatments (*p* ˂ 0.05) and days (*p* ˂ 0.05, Figure 2). OD values are displayed for collected exposure waters of each treatment (i.e., control, different solvents) for the 96 h periods before the water change (renewal period). There were no significant differences between NCs as well as between NCs and solvents after 96 h, apart from EtOH (*p* ˂ 0.05) and MeOH (*p* ˂ 0.05). OD in the EtOH treatment increased significantly within the first 24 h reaching its maximum at t = 24 h (*p* ˂ 0.05, Figure 2) followed by constantly declining values to reaching almost its initial value at t = 96 h. However, the OD data between t = 0 h and t = 96 h differed significantly for the EtOH (*p* < 0.05) and the 100µL L^−1^ of MeOH treatment (*p* ˂ 0.05), while no significant difference were found within other solvents and controls.

### 3.2. Coral Health Indicator on Organismal Level

#### 3.2.1. Photobiological Responses

**Figure 3:**
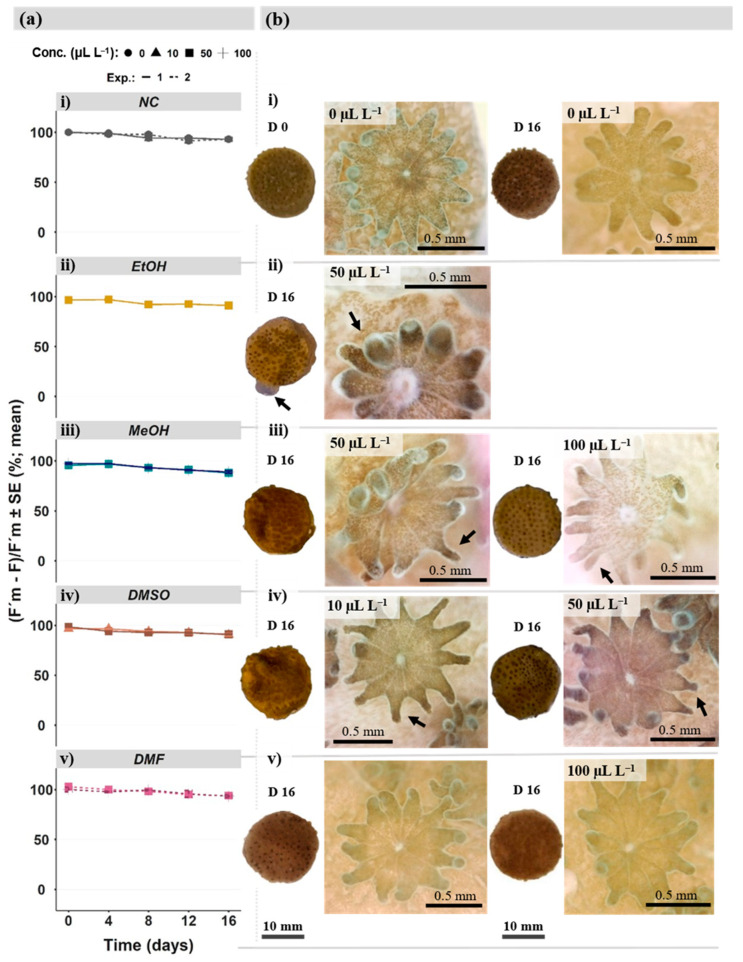
In (**a**), the symbionts effective photochemical efficiency of *Montipora digitata* are shown for 5 different time points (Day 0, 4, 8, 12, and 16) (i.e., control and solvent dose; (**i**–**v**)). (**b**) shows the images of one fragment at the beginning (**left**, D 0; 12 h after setting up the experiment) and the end of the exposure experiment (**right**, D 16) of each treatment, including related polyps (**right**) from day 16. Arrows indicate abnormalities in polyp development compared to the negative control (NC).

Effective photochemical efficiency ($\Phi_{PSII})$ data of *M. digitata* were displayed as mean values since it was normally distributed (W = 0.994; *p* = 0.797) and heterogenic (*p* < 0.05). There were no significant differences between control and treatments after 16 days of exposure (Figure 3a). No significant differences could be found for almost all treatments comparing day 0 with day 16, apart from the highest concentration (50 µL L^−1^) of DMSO. The $\left(\Phi_{PSII}\right)\;$declined significantly (*p* < 0.05) between day 0 and exposure day 16.

#### 3.2.2. Coral Fragment and Polyp Morphology

**Figure 4:**
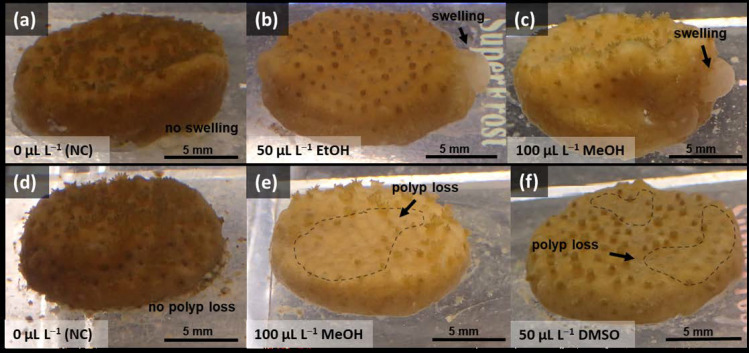
Examples of fragments of *Montipora digitata* with morphological abnormalities compared to the negative control (**a**,**d**), such as tissue swelling (**b**,**c**) and polyp losses (**e**,**f**) for different treatments after 16 days of exposure.

In Figure 3b, coral fragments (side/dorsal perspective) and polyps for control and each treatment were displayed. Examples of polyp abnormalities (e.g., polyp tentacle deformities) are indicated with arrows in Figure 3b, whereas fragment abnormalities (i.e., paleness, tissue swelling, polyp losses) were summarized in Table 1, with some examples highlighted in Figure 4a–f. Additional morphological information were collected in Supplementary Material Section S.2.1.

The morphological assessment of the control group at the end of the experiment (day 16) revealed no obvious difference compared to the test start (day 0). This was shown in Figure 3(bi). However, the control fragments were slightly paler at the end of the test (30%, Table 1) than at the beginning. Besides slightly paler fragments, only 1 of 24 fragments (4%) experienced polyp losses, and no tissue swelling occurred in any of the fragments (Table 1). Overall, the control fragments can be characterized as follows: homogeneous appearance with a dense polyp coverage (Figure 4a,d), pigmented polyps with homogeneously shaped tentacles, tentacles with rounded tips and fleshy, uniform appendages (Figure 3(bi)).

In contrast, the most apparent alterations in corals were found within the EtOH treatment (Figure 3(bii)). After 16 days, the fragments exposed to EtOH became 58% paler, accompanied with a 50% decline in polyp density (i.e., polyp losses) (Table 1). In addition, 66% of the fragments that experienced tissue alterations (Table 1) appeared with swelling tissue mostly at the side edge of the fragments, such as highlighted in Figure 4b. The EtOH treatment (Figure 3(bii)) resulted generally in smaller polyps with retracted and shorter tentacles, although the pigmentation was homogenously distributed. Effects of MeOH treatments (Figure 3(biii)) increased with concentration (i.e., 50 µL L^−1^; 100 µL L^−1^). Starting with paler fragments, increased polyp losses, and no tissue swelling in the lowest MeOH concentration, the highest concentration caused stronger fragment paleness, tissue alterations (i.e., tissue swelling; Figure 4c), and declining of polyp densities (Figure 3(biii); Figure 4e) (Table 1). At the polyp level, the highest MeOH concentration (Figure 3(biii)) resulted in greatly reduced pigmented, deformed, sharp, patchy, and distorted tentacles compared to the control. Similar patterns were observed for the two DMSO concentrations (i.e., 10 µL L^−1^; 50 µL L^−1^) (Figure 3(biv)). The lowest concentration resulted in 75% paler fragments and concentration-dependent increasing polyp losses (Table 1). For the highest concentration, fragment paleness and polyp losses occurred as severe as in the EtOH treatment (Table 1; Figure 4f), whereas tissue alterations (i.e., tissue swelling) could be found in 1 of 12 fragments (8%, Table 1). However, polyps revealed stronger tentacle deformities with sharper degraded tentacle tips at 10 µL L^−1^ and formed papules of tentacle tips at 50 µL L^−1^ DMSO (Figure 3(biv)). DMF resulted independently of the two concentrations (50 µL L^−1^; 100 µL L^−1^) in no effects compared to the control (Figure 3(bv)). Only polyp losses were found in 2 of the 12 fragments (Table 1).

### 3.3. Coral Health Indicator on Cellular Level

**Figure 5:**
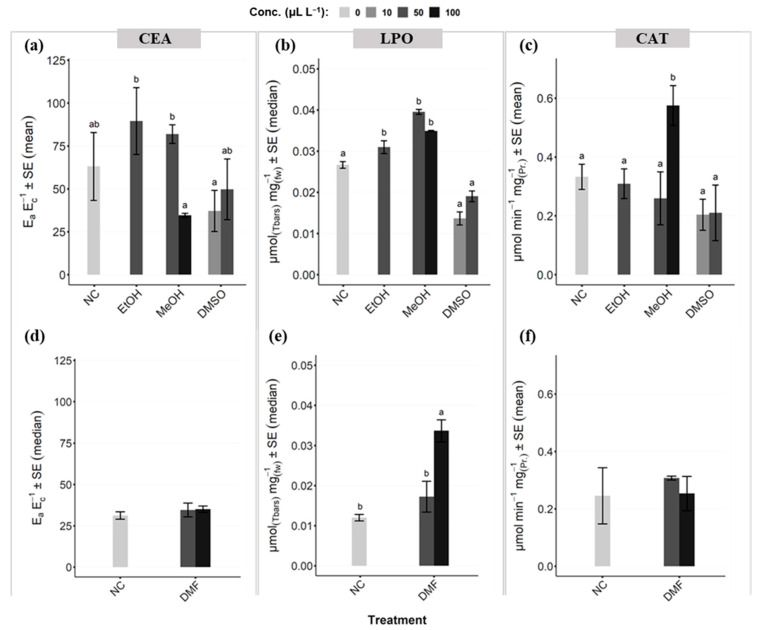
Biomarker analyses for *Montipora digitata* considering cellular energy allocation (CEA; (**a**,**d**)), lipid peroxidation (LPO, (**b**,**e**)), and catalase activity (CAT, (**c**,**f**)) for the control (NC) and each treatment. The biomarker analyses were separated in the two sub-experiments, whereas (**a**–**c**) described the first sub-experiment testing ethanol (EtOH), methanol (MeOH), and dimethyl sulfoxide (DMSO), and (**d**–**f**) the second sub-experiment testing dimethylformamide (DMF) of different concentrations including controls. Different letters (i.e., a,b,ab) between treatments indicate significances, while same letters indicating no significant differences. When no letters were shown in general, no differences occurred between any of the treatments.

The stress markers CEA, LPO, and CAT were displayed in mean or median values, depending on data distribution. For CEA (Figure 5a,d), there were no significant differences between the controls and the related treatments (i.e., solvent and concentrations) in each sub-experiment. However, in the first sub-experiment (Figure 5a) we could find significant differences (*p* < 0.05) between EtOH, and partly MeOH, to the lowest DMSO concentration. Within MeOH, we could additionally see a significant difference of the lowest (50 µL L^−1^) to the highest (100 µL L^−1^) concentration. No differences occurred between treatments in the second sub-experiment (Figure 5d). For LPO (Figure 5b,e), in both sub-experiments, we could find significant differences of treatments to the controls. In the first sub-experiment (Figure 5b), EtOH (50 µL L^−1^), and the two tested concentrations of MeOH (50 and 100 µL L^−1^) were significantly different to the control (*p* < 0.05), while we could not find any significant differences between the control and the two tested concentrations of DMSO (10 and 50 µL L^−1^). In the second experiment (Figure 5e), we could measure only for the highest DMF concentration (100 µL L^−1^) a significant difference (*p* < 0.05) to the control. At the same time, we could also find a significant difference (*p* < 0.05) between the two tested concentrations of DMF. In comparison, CAT showed only significant differences (*p* < 0.05) between the control and the highest MeOH concentration (100 µL L^−1^) of the first sub-experiment. Additionally, the effects of 100 µL L^−1^ MeOH differed significantly (*p* < 0.05) from 50 µL L^−1^ MeOH as well as the other solvents (Figure 5c). We could not detect any differences within the second sub-experiment (Figure 5f).

## 4. Discussion

The obtained data clearly showed that the tested solvent concentrations did not result in any mortality in *Montipora digitata* after 16 days of exposure. However, considering the choice of morphological and physiological endpoints, it became evident that *M. digitata* was affected even at solvent concentrations below those as recommended by the OECD guidance document entitled “Guidance document on aqueous-phase aquatic toxicity testing of difficult test chemicals” [16]. The response of the coral varied depending on the tested solvents and targeted concentrations. Overall, it can be concluded that the least and the highest effects were associated with DMF and EtOH, respectively. Based on sublethal and non-standardized criteria (i.e., photobiology, morphology, cell physiology), the solvents may be thus ranked as follows: DMF < DMSO ≈ MeOH ≤ EtOH.

The toxicity of DMF has been previously studied in marine algae (e.g., *Chlorella protothecoides*) [53] and invertebrates, such as *Daphnia magna* [54], with no observed effect concentrations (NOECs) at 500 to 1000 µL L^−1^. In fact, neither inhibition of growth, effects on reproduction, nor physiological effects have been reported. Similarly, 10 times lower concentrations (i.e., 50 or 100 µL L^−1^) of DMF in our study with the coral *M. digitata* did not detect any adverse effects at the organismal level. The photosynthetic efficiency of the coral symbionts as well as the morphological examination of the host indicated no abnormalities after 16 days of exposure. At the cellular level, physiological effects could be detected only for 100 µL L^−1^ DMF in LPO production, while CEA and CAT responses showed no significant differences. Higher production of LPO likely indicates higher production of toxic reactive oxygen species (ROS) such as free oxygen radicals (O_2_^─•^). The rise of free oxygen radicals may affect cell membranes components (i.e., unsaturated lipids) and initiate an auto-oxidation chain reaction resulting in cellular damage [55]. LPO can extensively increase when compounds such as lipid membranes are attacked by ROS resulting from various stresses. However, cell membranes are affected when ROS are overwhelming the threshold of various defending antioxidants, as outlined by Girotti [55] and Pannunzio and Storey [56]. Jyothi and his colleagues [57] highlighted the cellular toxicity of DMF-treated rats by an increased LPO production in various organs accompanied with enhanced organ damages. They suggested that DMF is downregulating antioxidative activities, which results in increased cellular damage (i.e., LPO production) by ROS. The study of Jyothi and his colleagues [57] provided a sense of how the use of DMF can adversely affect cell physiology (i.e., LPO production), although information from processes in aquatic organisms is sorely lacking.

Previous aquatic toxicity studies with DMSO on various algal species [15] or invertebrates [58] resulted in NOEC values of 400 to 1000 µL L^−1^. However, in our study, both DMSO concentrations (10 and 50 µL L^−1^) resulted in detectable morphological changes at the organismal level (i.e., tissue alterations, polyp abnormalities, polyp loss, strong polyp deformations), and affected the photosynthetic efficiency of the symbionts (the highest concentration only). Previous studies reported that DMSO can easily penetrate and diffuse into the cells or tissues [59,60], which might be the reason for its adverse effects on corals. When used as carrier solvent in ecotoxicological studies, these properties of DMSO could increase the membrane permeability of the tested substances that might lead to an over- or under-estimation of their toxicity. Furthermore, studies on cell physiology demonstrated that DMSO might destabilize cell membranes [59] by interacting with phospholipids, which as a consequence may lead to alterations in lipid packing of cell membranes and thus structural defects of membrane bilayers. The increased cell diffusion rate of DMSO could also result in the disruption of the water permeability of cells by blocking water movement through membranes, as already observed in several studies [61,62]. These observations also led to the assumption that DMSO acts as an agent for cell fusion or cell restructuring due to its destabilizing effects on membranes and by displacing water in cells [59]. Among other possibilities, this could serve as an explanation for the abnormal tentacle formation of polyps of *M. digitata* while exposed to DMSO. Yet, the DMSO concentrations assessed in these studies were up to 100 times higher than those we used in our study. In the absence of information on the concentration–response relationships of DMSO in corals cell physiology, we could assume that these effects (e.g., membrane permeability or destabilization) also occur at lower DMSO concentrations. The significant decrease in photosynthetic efficiency of symbionts in *M. digitata* exposed to 50 µL L^−1^ of DMSO might be explained by a surplus of DMSO affecting zooxanthella symbionts. Naturally, coral symbionts are exposed to ~10^−9^ µL L^−1^ of DMSO in marine habitat while they are able to oxidize DMSO to methanesulphonic acid (MSNA) as a potential ROS scavenging agent [63]. However, this process is accompanied by high energetic costs [64], so that an overload of DMSO, such as 50 µL L^−1^, might adversely affect their photo apparatus resulting in decreasing photosynthetic efficiency. However, on a cellular level, none of the selected cellular stress indicators (i.e., CEA, LPO, CAT) showed significant differences compared to the control. This was unexpected, since obvious morphological and, in some cases, photobiological effects were detected. The controversial findings for DMSO in detecting abnormal effects on organismal level, but no significant biochemical responses in any of the biomarkers, could be explained by its ability to scavenge, in particular, hydroxyl radicals (•OH) resulting from ROS production [65]. Klein, Cohen, and Cederbaum [65] showed that DMSO is degraded into formaldehyde by converting free active hydroxyl radicals which might control the formation of ROS and thus may reduce the LPO and CAT production in *M. digitata*. This also raises the question of how well biomarkers might be suitable in reflecting the overall health status in corals, when using DMSO as carrier solvent. However, its ability to scavenge stress-induced responses (i.e., ROS) or to alter membrane permeability could lead to the over- or under-estimation of the toxicity of hydrophobic compounds in toxicity studies.

Aquatic toxicological studies, which were performed for EtOH and MeOH on different marine organisms (e.g., algae, invertebrates) highlighted controversial finding. While the study with several algae species did show NOECs ranging from 14 to 10,000 µL L^−1^ for EtOH or 24–14,000 µL L^−1^ for MeOH [15], the NOEC for invertebrates such as *Daphnia magna* were located at around 10 µL L^−1^ [66] or for grass shrimps (i.e., *Palaemonetes pugio*) at 3000 µL L^−1^ [67] in EtOH treatments. Contrastingly, EtOH (i.e., 50 µL L^−1^) and MeOH (i.e., 50 and 100 µL L^−1^) in our study showed no significant changes in photosynthetic efficiency in *M. digitata* but revealed pronounced morphological effects, such as polyp and tissue abnormalities. In most of the exposed fragments, polyp losses occurred. It has been shown that polyp loss may be induced by severe stressors (e.g., unfavorable environmental conditions), and, in the case of polyp bailout, act as a defensive mechanism to evade such conditions [68]. We observed that coral fragments treated either with EtOH or MeOH showed tissue alterations such as tissue swelling and deformities. Tissue swelling has been reported by Cervino and colleagues [24], who studied the effects of different sodium cyanide (NaCN) concentrations (i.e., 50 to 600 mg L^−1^) on various stony corals. These authors related this abnormal behavior as a defensive and/or stress-related response; however, in their scenario the corals died at the end of the treatment. We also assume that the extremely punctuated and atypically swollen tissue of *M. digitata* in treatments with EtOH and MeOH (and partly DMSO), may be caused by defensive or stress-related cellular reactions. In addition, we observed an increase in seawater turbidity after 96 h (before the water change) with EtOH, and also to a lesser extent for the highest MeOH concentration (100 µL L^−1^). Smith and his colleagues [69] showed that EtOH (0.1 to 1%), compare to MeOH, has the ability to not only stimulate bacterial growth, but also acts as signaling molecule to change cell physiology in the test organism (i.e., *Acinetobacter* species), allowing the bacteria in their experiments to tolerate the toxic effects of salt. The rapid increase in turbidity during the EtOH treatment could therefore be explained by EtOH being metabolized by microorganisms, such as bacteria, and concurrent increase in its densities in the water ecosystems [33,70]. This can lead to problems in aquatic toxicology studies, such as the precipitation of hydrophobic test substances due to bacteria metabolizing the carrier solvents. In particular, OECD guidelines require that test compound concentrations should be maintained within 80–120% of nominal or mean measured values throughout the exposure period [16]. However, although we observed turbidity in MeOH to a lesser extent compared to EtOH in our experiments, other studies observed increased bacterial growth at higher MeOH concentrations [71,72]. The observed morphological abnormalities in MeOH-treated fragments may be also related to changes in the coral microbiome and may have induced stress in *M. digitata*. In this context Dinsdale, et al. [73] pointed out that perturbations in the coral environment, such as bacterial composition, can cause adverse effects (e.g., development of diseases and pathogens) that can lead to stress responses and even death of the coral.

At the cellular level, EtOH and MeOH resulted in significantly higher LPO production, compared to the control and DMSO. For comparison, both Downs, et al. [26] and Costa, et al. [74] observed higher LPO production in coral fragments exposed to short heat shock conditions or after being transplanted into new habitats and environments, respectively. The increased production of LPO in *M. digitata* exposed to alcoholic solvents confirms that *M. digitata* were more stressed in these treatments. However, Machado and colleagues [17] did not detect any significant differences of LPO production for a zoanthid (i.e., *Zoanthus* sp.) exposed to EtOH and MeOH in the concentration range of 10–2900 µL L^−1^ after 96 h exposure. This could be due to the production of higher levels of antioxidants, which may lead to a delayed LPO production phase [56,75]. The resistance of lipids to certain stressors and the organism’s antioxidant ability may influence the length of such a “lag phase” and is likely species specific [55]. Initial evidence of this was observed in the study performed by Costa, et al. [74]. Here, two soft coral species, namely *Sinularia polydactyla* and *Sinularia asterolobata*, responded differently to harsh stress events (i.e., coral shipping), resulting in *S. polydactyla* producing LPO, while *S. asterolobata* did not. A possible explanation could be the presence of effective antioxidants, such as mycosporine-glycine, which could inhibit LPO production [76]. Given these results, it is difficult to say whether biomarkers would be appropriate as a statistical endpoint for regulatory testing, as we may see inconsistent responses. This questions the suitability of such physiological endpoints in a standardized testing method if different coral species are used, as results might not be comparable between species.

However, the presence of different stressors stimulates oxidative stress and particular antioxidant responses in different groups of invertebrates. The main role of antioxidants such as CAT, is to neutralize the production of ROS by direct degradation [55,56]. The enzymatic degradation process of CAT, for example, is mostly described as part of the first antioxidant mechanisms phase [74]. CAT protect cells by degrading toxic hydrogen peroxide (H_2_O_2_) into water and oxygen that has been previously produced by enzymatic reaction of superoxide dismutase [56]. Previous studies highlighted increased CAT activities when organisms experience increased ROS levels induced by various stressors [74,77,78]. However, our study revealed that *M. digitata* did not show significant CAT activities compared to the controls, with the exception being the highest MeOH concentration (100 µL L^−1^) which exhibited high CAT responses in comparison. It seems that 100 µL L^−1^ of MeOH induced higher stress levels in *M. digitata*, resulting in high production of ROS and an enhanced CAT antioxidant defense reaction. This may be supported by the findings of Anithajothi, et al. [79], where higher CAT activity was positively associated with an increase in pathogenic- and stress-induced ROS production in several coral species. This could be supported by the slightly increased turbidity at the highest MeOH treatment in our study, which could indicate a change in the coral microbiome. Other studies indicated that increased CAT activity could be connected to enhanced metabolic activities as a result of the mobilization processes of reserves in cells under certain stresses (i.e., higher temperature) [80]. Higher stress levels due to high MeOH concentrations could therefore result in enhanced metabolic processes and the increase in CAT activities. However, information on the effect of MeOH on ROS production and corresponding increases of cellular antioxidant enzyme activities in corals is sorely lacking.

Nearly all of our physicochemical water parameters (see Supplementary Materials Section S.1.4) were maintained more than 80% within the acceptable ranges of parameters mainly recommended by Borneman [52] for in vitro coral husbandry (Supplementary Materials Section S.1.2). However, in the 96 h water semi–static renewal system, we could find a decline in nutrients (i.e., nitrate, phosphate) by more than 70% on average. Previous studies highlighted a correlation of imbalanced nutrient availability on the health of corals [81]. Phosphorus was described as the limiting factor and caused a brightening and disruption of the symbiosis (i.e., photosynthetic rate) when undersupplied. Conversely, corals were able to tolerate a depletion in nitrogen availability. In our study, *M. digitata* fragments experienced 30% paleness in the control group but no effects on the photosynthetic apparatus. Moreover, no abnormalities, either on the organismal or cellular level, could be found. A possible explanation for the strong decrease in nutrients such as nitrate, in particular, in combination with alcoholic solvents (i.e., EtOH) might be due to the increased proliferation of microorganisms [82]. The larger phosphate consumption, more drastic for 50 µL L^−1^ DMF, could be due to a higher uptake by the coral host and symbionts as a result of enhanced respiration acting as a phosphate sink [83]. Based on these results, we concluded that the nutrient decline in our control group was not detrimental to *M. digitata* over the course of the study.

## 5. Conclusions

Since solvents are commonly used in aquatic toxicity tests to facilitate the testing of hydrophobic or poorly water-soluble substances (e.g., UV filters, PHBs), knowledge of the intrinsic effects of such substances on the test organisms is crucial. This is of particular importance when non-standardized test organisms such as corals are used in combination with non-standardized (e.g., physiological, morphological) endpoints. Aside from substance-specific intrinsic effects, other aspects related to the use of carrier solvents, such as coral physiology (e.g., altering on cellular level) or water quality (e.g., increase in turbidity, utilization of the carrier solvent or changes in microbial composition), may also affect their health and/or substance availability and should be further investigated. When the use of solvents cannot be avoided in testing poorly water-soluble substances, our study showed that DMF was the most suitable solvent for testing the coral *M. digitata*. However, since physiological traits and stress resilience in corals are stretched across a vast range within the known species, one or a few representative corals species for such studies should be determined and established as model organisms. Given the variability in sensitivity to solvents among corals, additional solvent testing with proposed model corals should be performed. Investigation of both adverse and stress-related endpoints may be useful for a better understanding of coral physiology as well as pronounced adverse effects from a scientific perspective and could serve as a basis for further studies. Nevertheless, in a regulatory context the selection of biomarkers as a suitable toxicological sublethal endpoint requires further research.

## Figures and Tables

**Table 1:** Summary of morphological abnormalities (i.e., paleness, polyp losses, tissue swelling) on fragment level of *Montipora digitata*.

Treatment	Conc.	Total nr. of Fragments	Paleness		Polyp Losses		Tissue Swelling	
	µL L^−1^	TN	*n*	% of TN	*n*	% of TN	*n*	% of TN
NC	0	24	7	30	1	4	0	0
EtOH	50	12	7	58	6	50	8	66
MeOH	50	12	6	50	5	41	0	0
	100	12	9	75	5	41	4	33
DMSO	10	12	9	75	3	25	1	8
	50	12	8	66	6	50	1	8
DMF	50	12	2	16	2	16	0	0
	100	12	3	25	2	16	0	0

## Data Availability

The data presented in this study are available on request from the corresponding author.

## Supplementary Materials

The following supporting information can be downloaded at: https://www.mdpi.com/article/10.3390/toxics11040367/s1, Figure S1: Data of hobo pendant logger in incubator, for sub-experiment 1 (top) and 2 (bottom, black rectangle). Here, temperatures (red) and light intensities (blue) are displayed as line graphs, during 16 days of exposure. Hobo-logger position in the back of the incubator due to space limitation; Figure S2: Reference structure of fragments (left) and polyps (right) from day 1; Figure S3: Structures of fragments (left) and polyps (right) for NC (I) after 16 days of exposure; Figure S4: Structures of fragments (left) and polyps (right) for EtOH (50 µL L^−1^) after 16 days of exposure. Arrows indicating abnormalities; Figure S5: Structures of fragments (left) and polyps (right) for MeOH (50 µL L^−1^) after 16 days of exposure; Figure S6: Structures of fragments (left) and polyps (right) for MeOH (50 µL L^−1^) after 16 days of exposure; Figure S7: Structures of fragments (left) and polyps (right) for DMSO (10 µL L^−1^) after 16 days of exposure. Arrows indicating abnormalities; Figure S8: Structures of fragments (left) and polyps (right) for DMSO (50 µL L^−1^) after 16 days of exposure. Arrows indicating abnormalities; Figure S9: Structures of fragments (left) and polyps (right) for NC(II) after 16 days of exposure; Figure S10: Structures of fragments (left) and polyps (right) for DMF (50 µL L^−1^) after 16 days of exposure; Figure S11: Structures of fragments (left) and polyps (right) for DMF (100 µL L^−1^) after 16 days of exposure; Figure S12: Analysis of energy reserves for calculation of CEA for *M. digitata* considering carbohydrates (CBH; (a)), lipids (b) and protein (c) for the control (NC) and each treatment of the first sub-experiment. Here, same letters indicating no significant differences. No presented letters indicate no significant differences between treatments (i.e., control, solvent) at all; Figure S13: Analysis of energy consumption for calculation CEA for *M. digitata* considering energy transport system (ETS) for the control (NC) and each treatment of the first sub-experiment. Here, same letters indicating no significant differences; Figure S14: Analysis of energy reserves for calculation of CEA for *M. digitata* considering carbohydrates (CBH; (a)), lipids (b), and protein (c) for the control (NC) and each treatment of the second sub-experiment. Here, same letters indicating no significant differences. No presented letters indicate no significant differences between treatments *(*i.e., control, solvent) at all; Figure S15: Analysis of energy consumption for calculation CEA for *M. digitata* considering energy transport system (ETS) for the control (NC) and each treatment second sub-experiment. Here, same letters indicating no significant differences; Table S1: Main husbandry parameters in the aquarium facility of the ICBM; Table S2: The main physico-chemical parameters and their ranges for the maintenance of corals in closed aquarium systems compared to natural conditions in coral reefs, modified from Borneman [1/53] (SSI = sea surface irradiation); Table S3: Measured average water parameters within the 96 h water renewal interval; Table S4: Static analysis for each measured marker (i.e., optical density, photosynthetic efficiency) (grey background = comparisons with controls; bold = indicating statistical significances); Table S5: Static analysis for each measured biomarker (i.e., cellular energy allocation, lipid peroxidation, catalyze) of each sub-experiment. (grey background= comparisons with controls; bold = indicating statistical significances); Table S6: Static analysis for energy reserves (i.e., carbohydrates, lipids, protein) and energy consumption (i.e., energy transport system) for CEA calculations of each sub-experiment (grey background = comparisons with controls; bold = indicating statistical significances). References [52,84,85] are cited in the supplementary materials.
