# Supplementary material for: Ecotoxicological Effects of Four Commonly Used Organic Solvents on the Scleractinian Coral Montipora digitata
Source: Toxics. 2023 Apr 12;11(4):367. doi: 10.3390/toxics11040367 (PMC10146860; doi:10.3390/toxics11040367)
Supplement: Supplementary file 1 [file toxics-11-00367-s001.zip › toxics-2319627-supplementary.pdf]

Supplementary material for

Ecotoxicological effects of four commonly used organic solvents on the scleractininan coral *Montipora digitata*

Valentina Di Mauro1*, Elham Kamyab[2], Matthias Y. Kellermann[1], Mareen Moeller[1], Samuel Nietzer[1], Laura H. Lüt-jens[3], Sascha Pawlowski[3], Mechtild Petersen-Thiery[4], Peter J. Schupp[1,5*]

[1] Environmental Biochemistry, Institute for Chemistry and Biology of the Marine Environment (ICBM), School of Mathematics and Science, Carl von Ossietzky University of Oldenburg, Schleusenstr. 1, 26382 Wilhelmshaven, Germany
[2] German Center for Marine Biodiversity Research (DZMB), Senckenberg am Meer, 26382 Wilhelmshaven, Germany
[3] Department of Product Safety, Regulatory Ecotoxicology, BASF SE, Carl-Bosch-Str. 38, 67056 Ludwigshafen am Rhein, Germany
[4] Product Stewardship & EHS Data Management, BASF Personal Care and Nutrition GmbH, Rheinpromenade 1, 40789 Monheim am Rhein, Germany
[5] Helmholtz Institute for Functional Marine Biodiversity (HIFMB) at the University of Oldenburg (HIFMB), Ammerländer Heerstr. 231, 26129 Oldenburg, Germany

* Correspondence: Valentina Di Mauro, valentina.di.mauro@uni-oldenburg.de; Peter J. Schupp, peter.schupp@uni-oldenburg.de

[*] Corresponding authors:

Valentina Di Mauro, valentina.di.mauro@uni-oldenburg.de

Peter J. Schupp, peter.schupp@uni-oldenburg.de

S1 – Material and Method

S.1.1. Husbandry conditions (water parameters) in the aquarium facility of the Institute for Chemistry and Biology of the Marine Environment (ICBM, Wilhelmshaven, Germany)

Table S1. Main husbandry parameters in the aquarium facility of the ICBM.

Physical-chemical parameter	Unit	In facility
		aquarium
Temperature	°C	26 ± 0.5
Salinity	PSU	34 - 35
Alkalinity	meg L^{-1}	2.5 - 2.7
O_2		> 75% saturation
pH		8.00 - 8.35
Calcium	mg L^{-1}	400 - 440
Magnesium	mg L^{-1}	1300 - 1380
Nitrate	mg L^{-1}	2 - 5
Phosphate	mg L^{-1}	0.06 - 0.15

S.1.2. Threshold of water parameters in coral husbandry

Table S2: The main physico-chemical parameters and their ranges for the maintenance of corals in closed aquarium systems compared to natural conditions in coral reefs, modified from Borneman [1] (SSI = sea surface irradiation). [a] Minimum values as recommended by Fosså and Nilsen [2]. [b] Recommended range by TropicMarin [3]

Physical-chemical parameter	Unit	Range in reefs	Acceptable range	Ideal range	Importance
		nature	*aquarium*	*aquarium*	
Light	µE m^2	40 - 10% SSI	30 - 10% SSI	> 10% SSI	critical
Temperature	°C	21 - 30	24 - 28	26 - 28	critical
Salinity	PSU	23 - 40	33 - 38	24 - 36	critical
Alkalinity	mg L^{-1}	2.0 - 2.5	2.0 - 4.5	3.5 - 4.0	critical
O_2		highly variable	slightly variable	> 75% saturation	critical
pH		*7.4 - 8.4*	*7.8 - 8.8*	*8.2 - 8.6*	important
Calcium	mg L^{-1}	*400-430*	*350-500*	*425 - 450*	important
Nitrate	mg L^{-1}	*0.00 - 0.20*	*< 10.00*	*0.00 - 1.00*	important
Phosphate	mg L^{-1}	*0.00 - 0.02*	*> 0.015*[a]	*0.03 - 0.1*[b]	essential

S.1.3. Monitoring (HOBO Logger data) of incubator conditions during 16 days of experiment

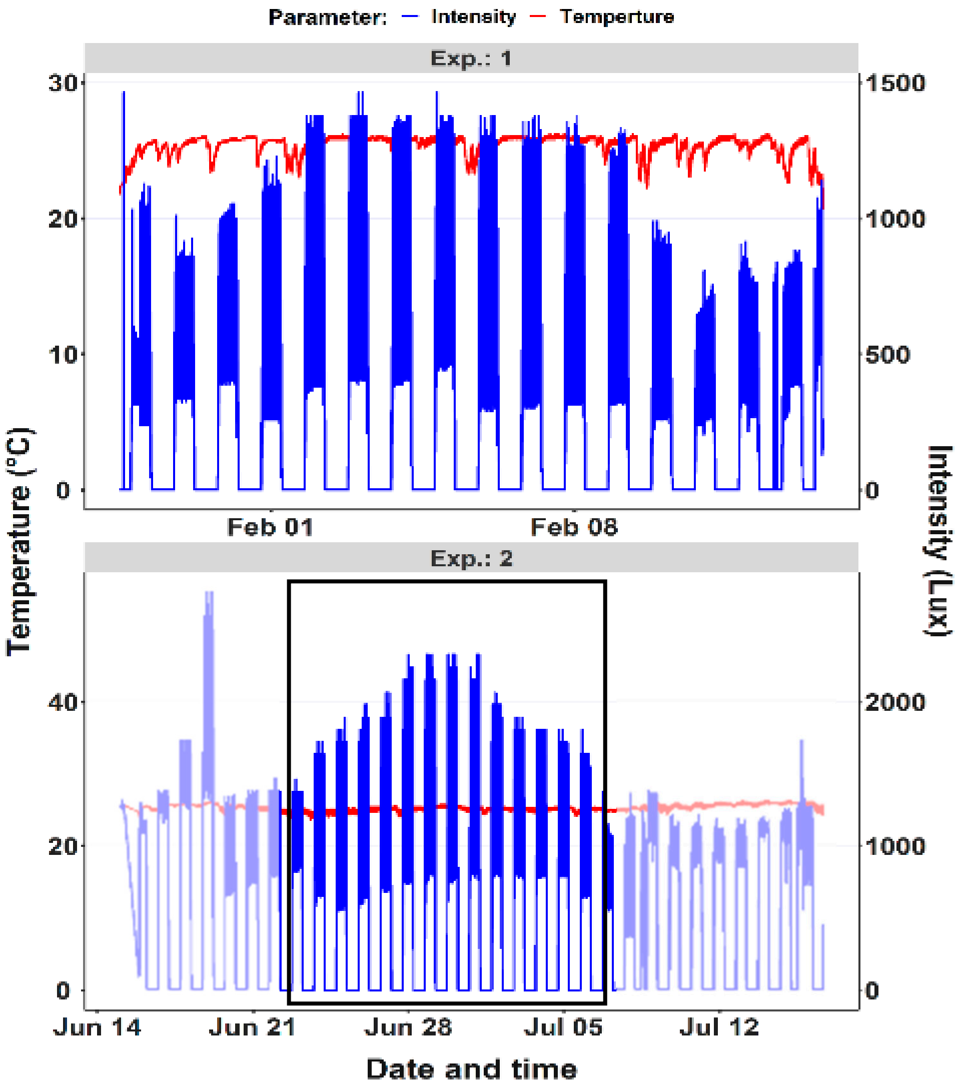

Figure S1: Data of hobo pendant logger in incubator, for sub-experiment 1 (top) and 2 (bottom, black rectangle). Here, temperatures (red) and light intensities (blue) are displayed as line graphs, during 16 days of exposure. Hobo-logger position in the back of the incubator due to space limitation.

S.1.4. Measured water parameters during 16 days of exposure:

Table S3: Measured average water parameters within the 96-h water renewal interval.

Treatment	Dose in µl L^{-1}	Sampling point in h	100% water renewal intervals	Nr. of replicates	N	Sal in PSU		O_2 in mg L^{-1}		pH		Ca in mg L^{-1}		ALK in meg L^{-1}		PO_4 mg L^{-1}		NO_3 mg L^{-1}	
						Value	SE	Value	SE	Value	SE	Value	SE	Value	SE	Value	SE	Value	SE
Initial: 0.2 µm filtered artificial seawater media	0	0	flow through system	-	8	33.90	0.07	7.250	0.165	8.10	0.02	454.0	7.5	2.82	0.04	0.133	0.014	1.441	0.313
Negative Control	0	96	4	6	8	35.53	0.28	6.754	0.043	7.89	0.29	449.5	6.2	2.50	0.08	0.037	0.017	0.191	0.104
Ethanol	50	96	4	3	4	35.56	0.27	6.668	0.051	8.06	0.03	450.1	12.5	2.32	0.10	0.044	0.005	0.068 *	0.031
Methanol	50	96	4	3	4	36.43	0.30	6.591	0.185	8.06	0.06	464.2	6.4	2.50	0.12	0.066	0.040	0.273	0.245
Methanol	100	96	4	3	4	36.98	0.26	6.493	0.242	8.06	0.05	455.2	14.8	2.45	0.12	0.072	0.037	0.200	0.160
Dimethyl sulfoxide	10	96	4	3	4	36.96	0.13	6.675	0.184	8.07	0.03	460.4	12.5	2.36	0.10	0.037	0.007	0.474	0.233
Dimethyl sulfoxide	50	96	4	3	4	36.83	0.65	6.713	0.067	8.09	0.04	469.3	8.5	2.45	0.22	0.039	0.011	0.339	0.233
Dimethyl formamide	50	96	4	3	4	35.93	0.21	6.643	0.035	8.24	0.02	441.2	10.7	2.62	0.03	0.003 *	0.001	0.197	0.098
Dimethyl formamide	100	96	4	3	4	35.31	0.19	6.615	0.047	8.24	0.02	436.1	7.9	2.67	0.10	0.015	0.012	0.278	0.156
**Ø** total (t0; t96)	-	-	-	-	9	35.94	0.33	6.711	0.072	8.09	0.03	453.3	3.5	2.52	0.05	0.050	0.013	0.385	0.137
**% recovery of initial value after 96 h:**						**106.8**		**91.6**		**99.8**		**99.8**		**88.1**		**29.4**		**17.5**	

(*) values with strongest variation to related data-set

Internal

S2 – Results

S2.1 – Morphology

S2.1.1. Reference from day 1

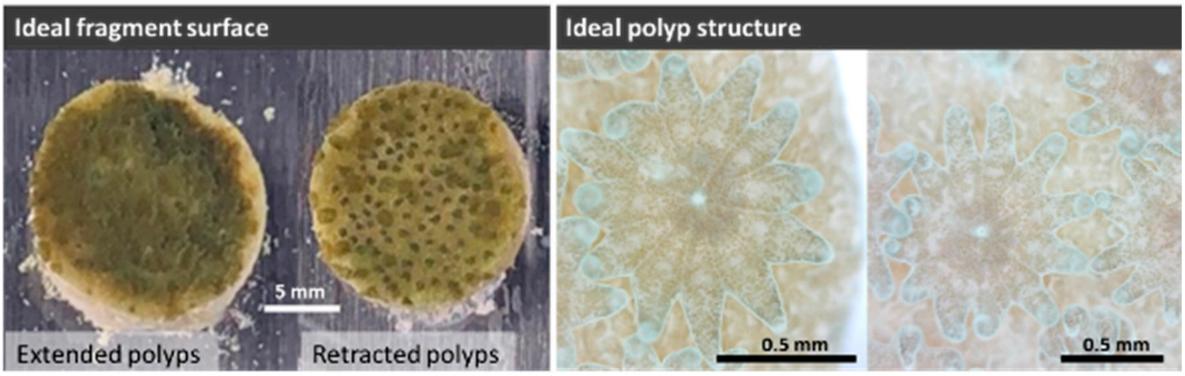

Figure S2: Reference structure of fragments (left) and polyps (right) from day 1.

Reference structure:

- Fragments (left): Polyps on fragment surface are homogenously distributed and dense; saturated colour, even when polyps are retracted
- Polyps (right): round shape, well pigmented, round fleshy tentacles

S.2.1.2. First sub-experiment (Exp.:1)

S.2.1.2.1. Negative control (NC I) at day 16

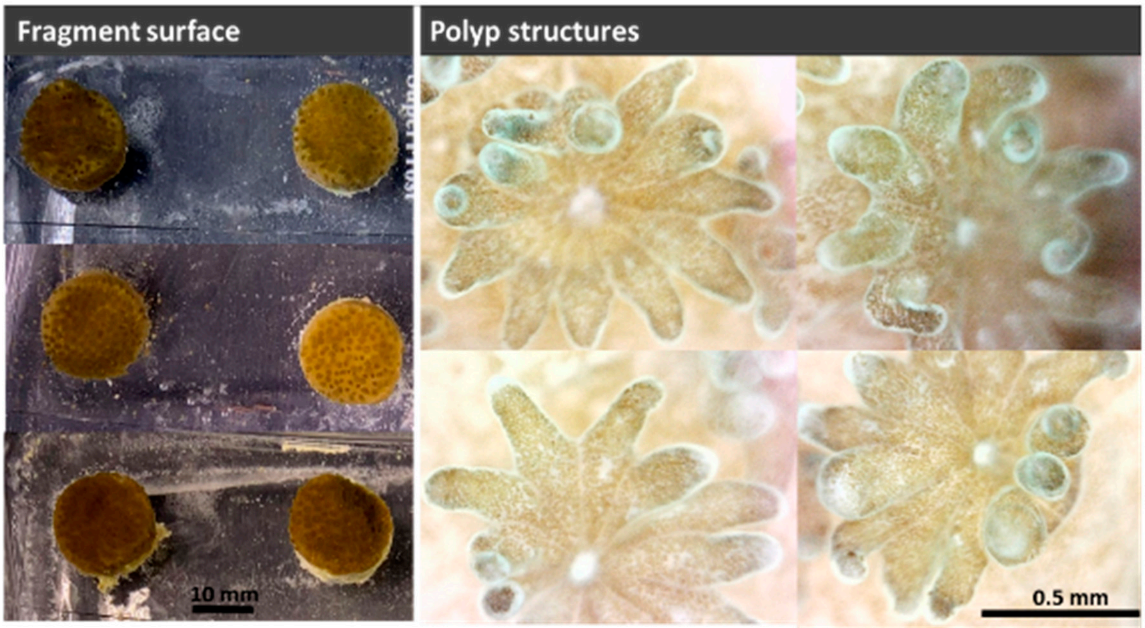

Figure S3: Structures of fragments (left) and polyps (right) for NC(I) after 16 days of exposure.

NC structure:

- Fragments (left): Polyps on fragment surface are homogenously distributed and dense; saturated colour, even when polyps are retracted; occasionally slightly brighter
- Polyps (right): round shape, well pigmented, uniform round fleshy tentacles

S.2.1.2.2. 50 µL L^{-1} ethanol (EtOH) at day 16

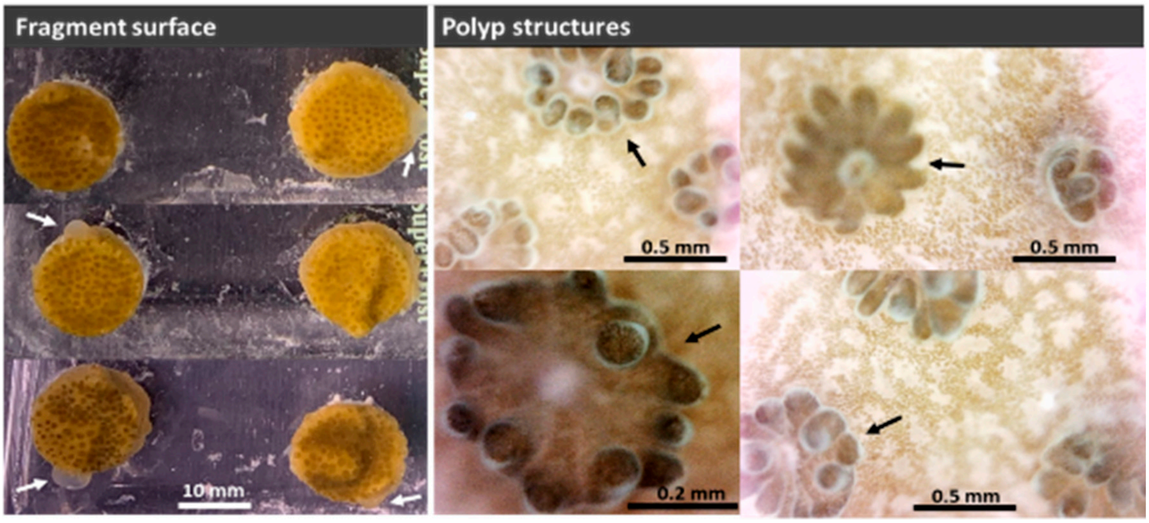

Figure S4: Structures of fragments (left) and polyps (right) for EtOH (50 µL L^{-1}) after 16 days of exposure. Arrows indicating abnormalities.

EtOH structure:

- Fragments (left): Polyps on fragment are well distributed; occasionally losses of polyps; mostly slightly brighter; tissue alterations (swelling) indicated with white arrows
- Polyps (right): round but small shape, maintained pigmentation, retracted tentacles (black arrows)

S2.1.2.3. 50 µL L^{-1} methanol (MeOH 50) at day 16

MeOH 50 structure:

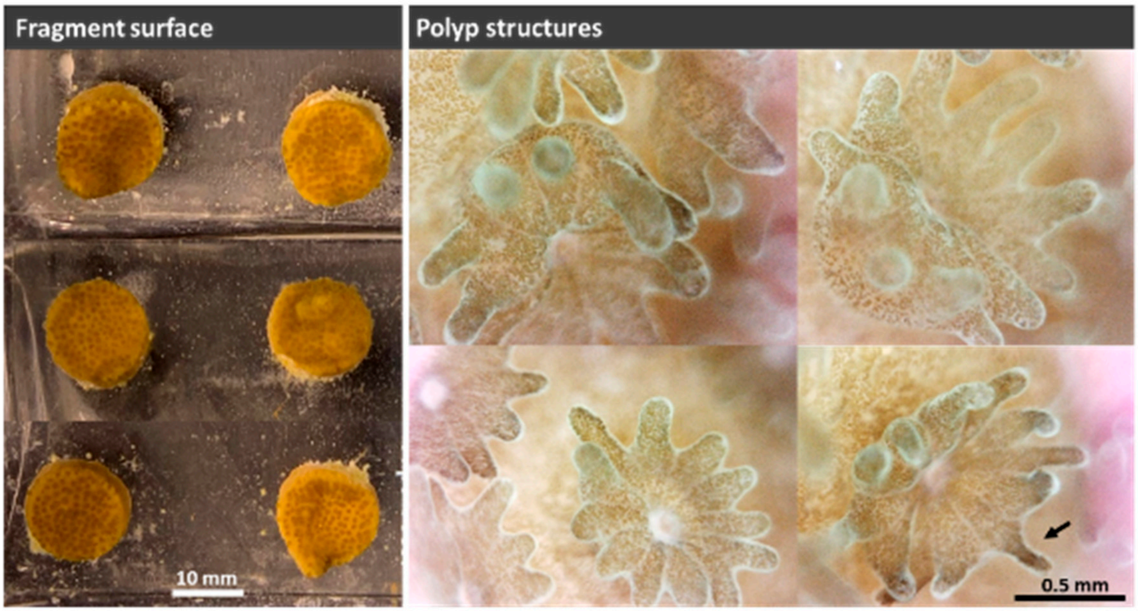

Figure S5: Structures of fragments (left) and polyps (right) for MeOH (50 µL L^{-1}) after 16 days of exposure.

- Fragments (left): Polyps on fragment are well distributed; occasionally losses of polyps; brighter fragments
- Polyps (right): round shape, well pigmented; round, fleshy but occasionally more retracted tentacles; occasional abnormalities (black arrow)

S.2.1.2.4. 100 µL L^{-1} methanol (MeOH 100) at day 16

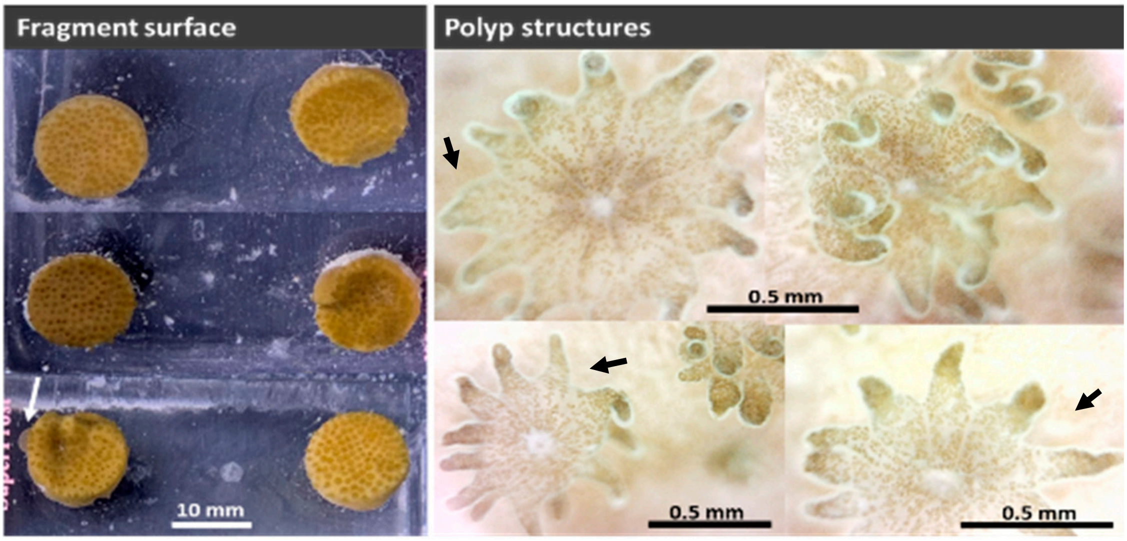

Figure S6: Structures of fragments (left) and polyps (right) for MeOH (100 µL L^{-1}) after 16 days of exposure. Arrows indicating abnormalities.

MeOH 100 structure:

- Fragments (left): Polyps on fragment are well distributed; occasionally losses of polyps; mostly slightly brighter; tissue alterations (swelling) indicated with white arrows
- Polyps (right): round, but punctuated pigmentation; abnormal sharp, patchy and distorted tentacles (black arrows)

S2.1.2.5. 10 µL L^{-1} dimethyl sulfoxide (DMSO 10) at day 16

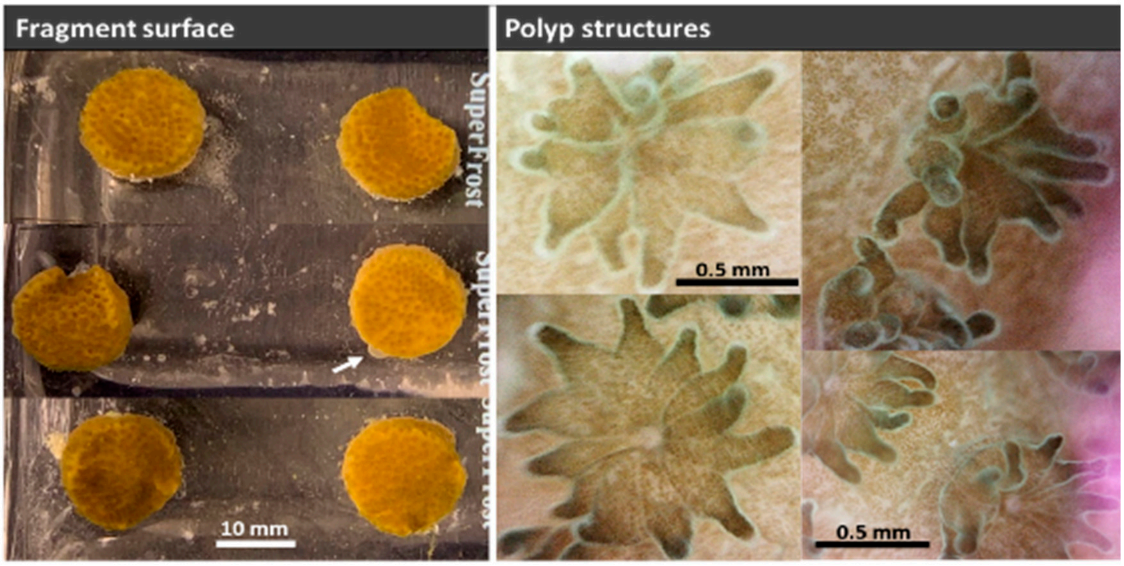

Figure S7: Structures of fragments (left) and polyps (right) for DMSO (10 µL L^{-1}) after 16 days of exposure. Arrows indicating abnormalities.

DMSO 10 structure:

- Fragments (left): Polyps on fragment are well distributed; occasionally losses of polyps; mostly slightly brighter; occasionally tissue alterations (swelling) indicated with white arrows
- Polyps (right): round; stronger pigmentation especially in tips; abnormal patchy and distorted tentacles, with nodular tips (black arrows)

Internal

S2.1.2.6. 50 µL L^{-1} dimethyl sulfoxide (DMSO 50) at day 16

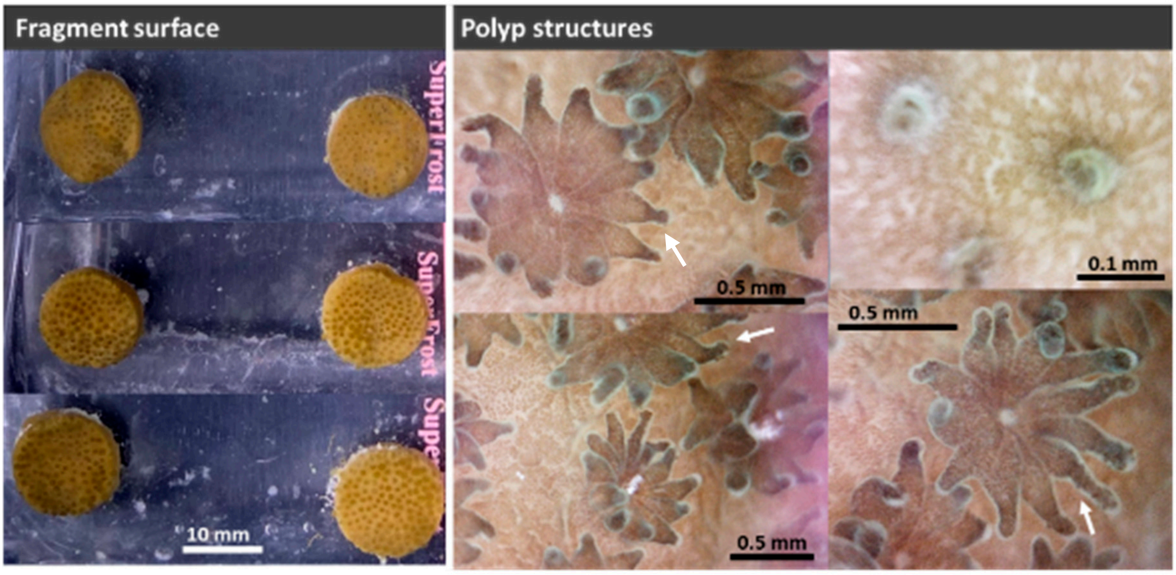

Figure S8: Structures of fragments (left) and polyps (right) for DMSO (50 µL L^{-1}) after 16 days of exposure. Arrows indicating abnormalities.

DMSO 50 structure:

- Fragments (left): Polyps on fragment are well distributed; occasionally losses of polyps; mostly slightly brighter
- Polyps (right): round; stronger pigmentation especially in tips; strong abnormal, patchy and distorted tentacles, with nodular tips (white arrows)

S2.1.3. Second sub-experiment (Exp.: 2)

S2.1.3.1. Negative control (NC II) at day 16

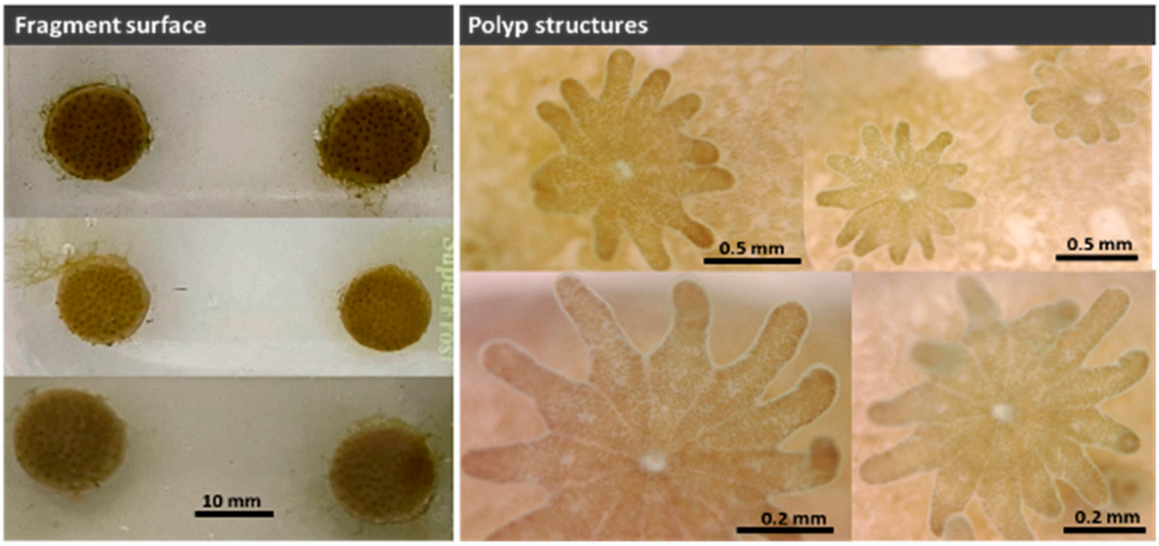

Figure S9: Structures of fragments (left) and polyps (right) for NC(II) after 16 days of exposure.

NC structure:

- Fragments (left): Polyps on fragment surface are homogenously distributed and dense; saturated colour, even when polyps are retracted; occasionally slightly brighter
- Polyps (right): round shape, well pigmented, uniform round fleshy tentacles

S2.1.3.2. 50 µL L^{-1} dimethyl formamide (DMF 50) at day 16

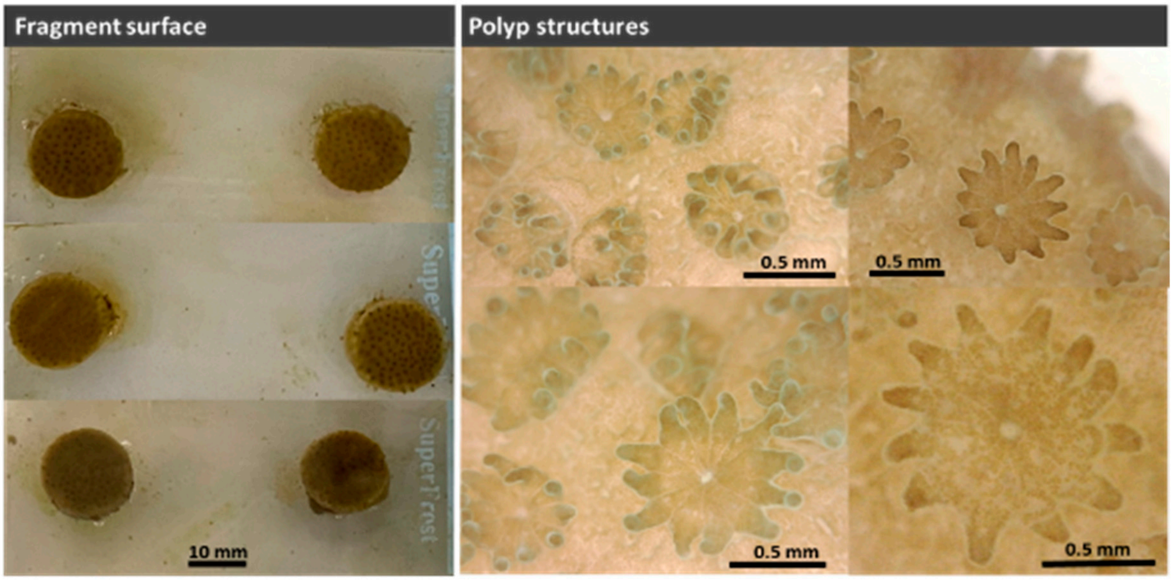

Figure S10: Structures of fragments (left) and polyps (right) for DMF (50 µL L^{-1}) after 16 days of exposure.

DMF 50 structure:

- Fragments (left): Polyps on fragment surface are homogenously distributed and dense; saturated colour, even when polyps are retracted
- Polyps (right): round shape, well pigmented; uniform round, fleshy and extended tentacles

S2.1.3.3. 100 µL L^{-1} dimethyl formamide (DMF 100) at day 16

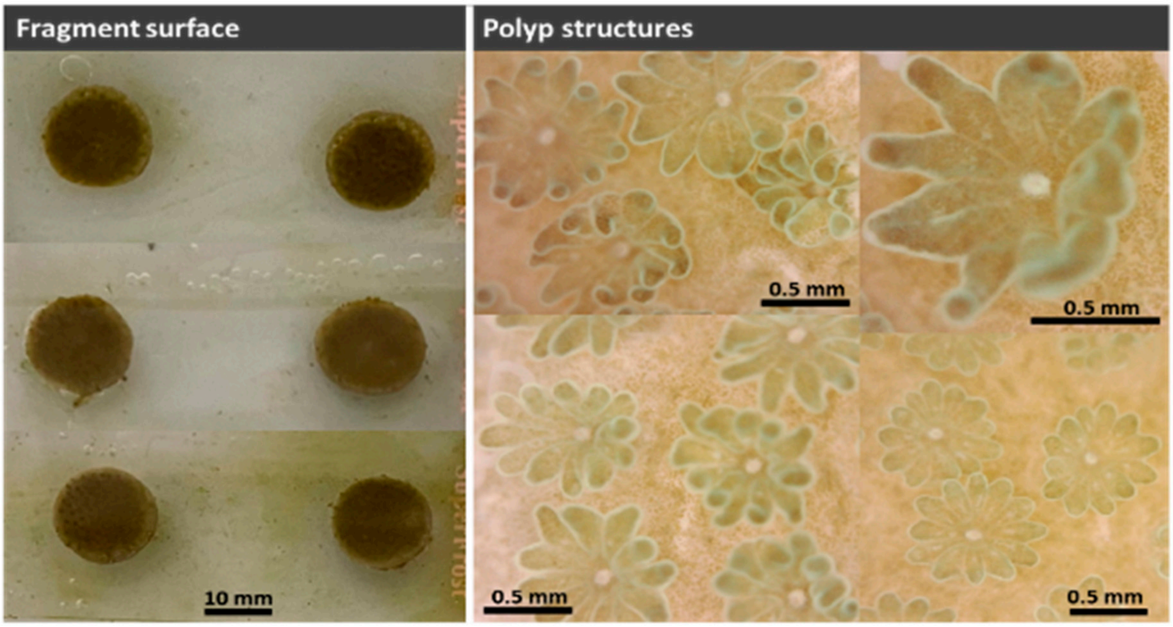

Figure S11: Structures of fragments (left) and polyps (right) for DMF(100 µL L^{-1}) after 16 days of exposure

DMF 100 structure:

- Fragments (left): Polyps on fragment surface are homogenously distributed and still dense; saturated colour, even when polyps are retracted
- Polyps (right): round shape, well pigmented; uniform round, fleshy and extended tentacles

S.2.2. Biomarker cellular energy allocation as it is calculated form energy availability (i.e., carbohydrates (CBH), lipids, proteins) and energy consumption (i.e., energy transport system (ETS))

S.2.2.1 – Sub-experiment 1

- ***Energy availability:***

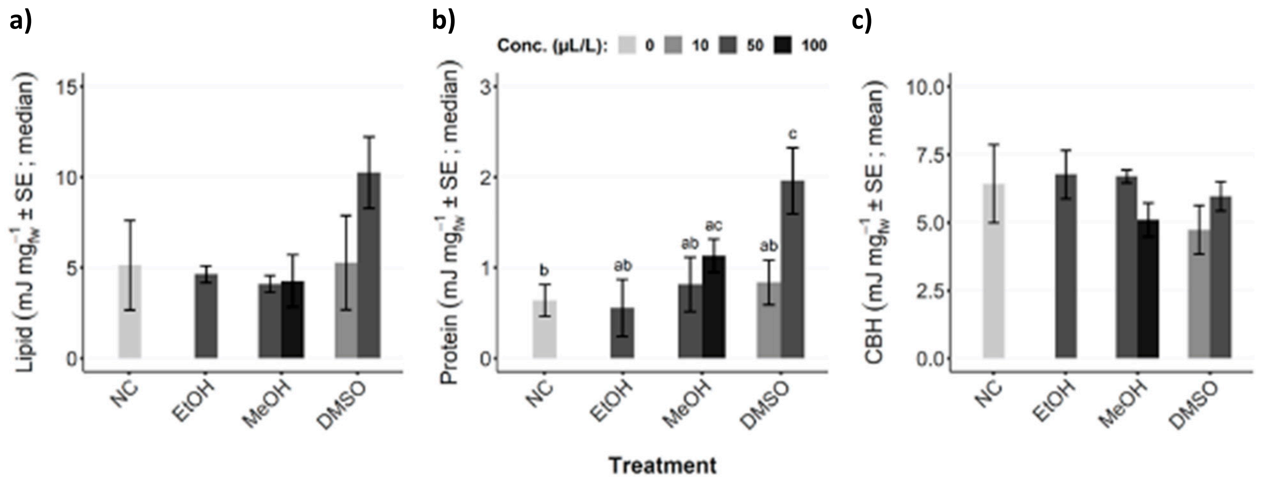

Figure S12: Analysis of energy reserves in respect of calculating CEA for M. digitata considering carbohydrates (CBH; (a)), lipids (b) and protein (c) for the control (NC) and each treatment of the first sub-experiment. Here, same letters indicating no significant differences. No presented letters indicate no significant differences between treatments (i.e., control, solvent) at all.

- ***Energy consumption:***

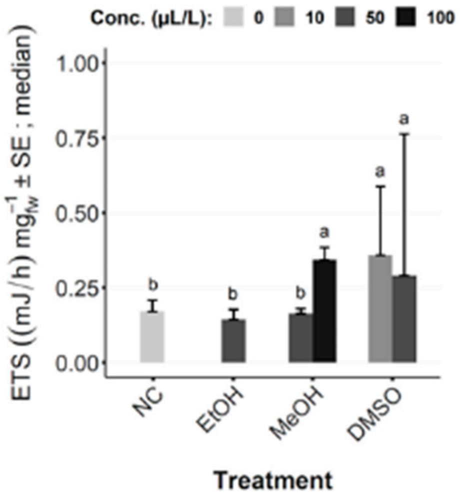

Figure S13: Analysis of energy consumption in respect of calculating CEA for M. digitata considering energy transport system (ETS) for the control (NC) and each treatment of the first sub-experiment. Here, same letters indicating no significant differences.

S.2.2.1 – Sub-experiment 2

- ***Energy availability:***

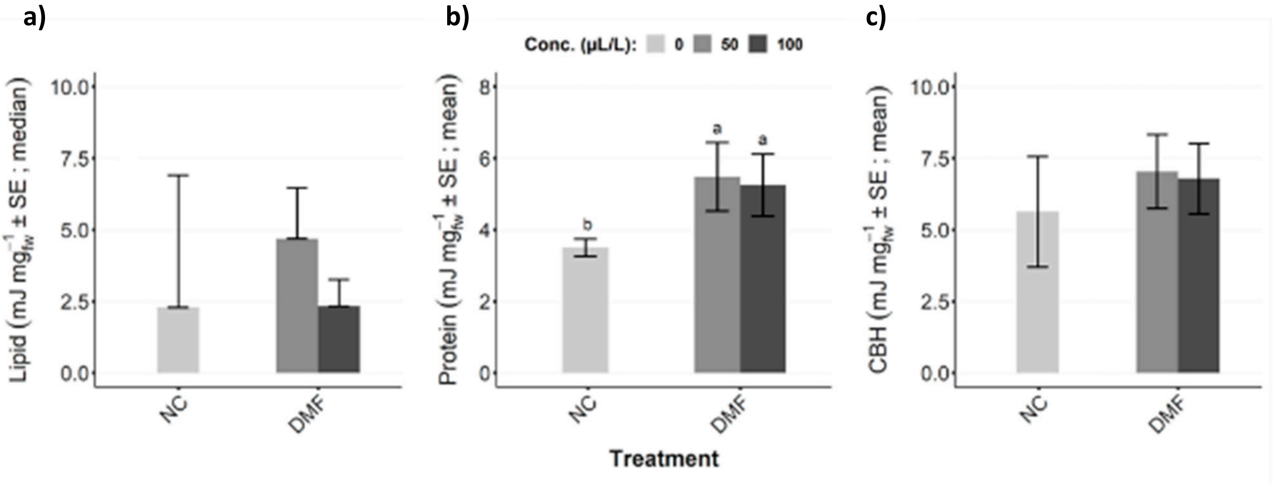

Figure S14: Analysis of energy reserves in respect of calculating CEA for M. digitata considering carbohydrates (CBH; (a)), lipids (b) and protein (c) for the control (NC) and each treatment of the second sub-experiment. Here, same letters indicating no significant differences. No presented letters indicate no significant differences between treatments (i.e., control, solvent) at all.

- ***Energy consumption:***

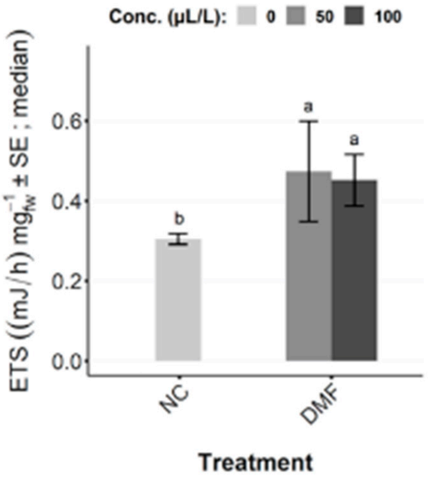

Figure S15: Analysis of energy consumption in respect of calculating CEA for M. digitata considering energy transport system (ETS) for the control (NC) and each treatment second sub-experiment. Here, same letters indicating no significant differences.

S3 – Statistics

S.3.1. Statistics for optical density and photosynthetic efficiency of the two sub-experiments

Table S4: Statistical analysis for each measured marker (i.e., optical density, photosynthetic efficiency) (grey background= comparisons with controls; bold= indicating statistical significances).

Marker	*Statistical Test*	*Comparison*					*Statistic*	*p-value*
Optical density (OD)								
	Shapiro-Wilk normality test						*W=0.51141*	*<0.0001*
	Levene's Test for homogeneity of variance (centre = median)						*F=1.4917*	*0,0089*
	Kruskal-Wallis rank sum test						*$\chi 2$ (63)=329.44*	*<0.0001*
		Group1	***t(d)***	***-***	***Group2***	***t(d)***		
	Dunn´s post hoc comparison	*NC1*		*-*	*NC2*		*-0,8318*	*0,5615*
		NC1		***-***	***EtOH***		***-7,4152***	***<0.0001***
		NC1		*-*	*MeOH 50*		*1,2448*	*0,3688*
		NC1		*-*	*MeOH 100*		*-1,717*	*0,1821*
		NC1		*-*	*DMSO 10*		*0,527*	*0,6602*
		NC1		*-*	*DMSO 50*		*-0,6601*	*0,6321*
		NC2		*-*	*DMF 50*		*-1,5507*	*0,242*
		NC2		*-*	*DMF 100*		*-2,068*	*0,1071*
		EtOH 50		***-***	***MeOH 50***		***-8,66***	***<0.0001***
		EtOH 50		***-***	***MeOH 100***		***-5,6834***	***<0.0001***
		EtOH 50		***-***	***DMSO 10***		***7,9423***	***<0.0001***
		EtOH 50		***-***	***DMSO 50***		***6,7406***	***<0.0001***
		EtOH 50		***-***	***DMF 50***		***6,8501***	***<0.0001***
		EtOH 50		***-***	***DMF 100***		***6,3422***	***<0.0001***
Photosynthetic* efficiency *(PAM)								
	Shapiro-Wilk normality test						*0,9935*	*0,7974*
	Bartlett test of homogeneity of variances						*k^2= 67.265*	*0,01353*
	Welch ANOVA						*17,9*	*<0.0001*
	Games Howell post hoc comparison	*NC1*	*0*	*-*	*NC2*	*0*	*0,000*	*1,000*
		NC1	*16*	*-*	*NC2*	*16*	*0,190*	*1,000*
		NC1	*16*	*-*	*EtOH*	*16*	*1,920*	*1,000*
		NC1	*16*	*-*	*MeOH 50*	*16*	*5,001*	*0,828*
		NC1	*16*	*-*	*MeOH 100*	*16*	*4,072*	*0,936*
		NC1	*16*	*-*	*DMSO 10*	*16*	*2,364*	*1,000*
		NC1	*16*	*-*	*DMSO 50*	*16*	*1,899*	*0,999*
		NC2	*16*	*-*	*DMF 50*	*16*	*-0,814*	*1,000*
		NC2	*16*	*-*	*DMF 100*	*16*	*-0,207*	*1,000*
		NC1	*0*	*-*	*NC1*	*16*	*-7,067*	*0,454*
		NC2	*0*	*-*	*NC2*	*16*	*-6,877*	*0,118*
		EtOH	*0*	*-*	*EtOH*	*16*	*-5,558*	*0,542*
		MeOH 50	*0*	*-*	*MeOH 50*	*16*	*-7,571*	*0,208*
		MeOH 100	*0*	*-*	*MeOH 100*	*16*	*-8,485*	*0,141*
		DMSO 10	*0*	*-*	*DMSO 10*	*16*	*-5,840*	*0,394*
		DMSO 50	***0***	***-***	***DMSO 50***	***16***	***-7,384***	***0,033***
		DMF 50	*0*	*-*	*DMF 50*	*16*	*-8,897*	*0,167*
		DMF 100	*0*	*-*	*DMF 100*	*16*	*-6,807*	*0,718*

Internal

S.3.2 Statistics for biomarker analyses (i.e., CEA, LPO, CAT) of the two sub-experiments.

Table S5: Statistical analysis for each measured biomarker (i.e., cellular energy allocation, lipid peroxidation, catalyse) of each sub-experiment. (grey background= comparisons with controls; bold= indicating statistical significances)

Biomarker	Statistical Test	Group1	-	Group2	Statistic	p-value
**SUB-EXPERIMENT 1**						
**Cellular energy allocation (CEA)**						
	Shapiro-Wilk normality test				W=0.96145	0.08
	Bartlett test of homogeneity of variances				k^2=35.124	<0.0001
	Welch ANOVA				F=18.165	<0.0001
	Games Howell post hoc comparison	Group1	-	Group2		
		NC1	-	EtOH	-26.447	0.512
		NC1	-	MeOH 50	-18.866	0.612
		NC1	-	MeOH 100	28.484	0.165
		NC1	-	DMSO 10	25.936	0.339
		NC1	-	DMSO 50	13.333	0.924
		EtOH	-	MeOH 50	-7.580	0.987
		**EtOH**	**-**	**MeOH 100**	**-54.931**	**0.008**
		**EtOH**	**-**	**DMSO 10**	**52.382**	**0.012**
		EtOH	-	DMSO 50	39.780	0.111
		**MeOH 50**	**-**	**MeOH 100**	**47.351**	**0.000**
		**MeOH 50**	**-**	**DMSO 10**	**44.802**	**0.001**
		MeOH 50	-	DMSO 50	32.199	0.089
		MeOH 100	-	DMSO 10	-2.549	0.999
		MeOH 100	-	DMSO 50	-15.151	0.601
		DMSO 10	-	DMSO 50	12.603	0.872
**Lipid peroxidation (LPO)**						
	Shapiro-Wilk normality test				W= 0.9508	0.02695
	Levene's Test for Homogeneity of Variance (center = median)				F= 0.9751	0.4426
	Kruskal-Wallis rank sum test				χ2 (5)= 39.542	<0.0001
	Dunn´s post hoc comparison	**NC1**	**-**	**EtOH**	**-2.427**	**0.029**
		**NC1**	**-**	**MeOH 50**	**-2.187**	**0.048**
		**NC1**	**-**	**MeOH 100**	**-2.802**	**0.011**
		NC1	-	DMSO 10	1.753	0.119
		NC1	-	DMSO 50	1.214	0.307
		EtOH	-	MeOH 50	-0.240	0.811
		EtOH	-	MeOH 100	0.375	0.759
		**EtOH**	**-**	**DMSO 10**	**4.180**	**0.000**
		**EtOH**	**-**	**DMSO 50**	**3.641**	**0.001**
		MeOH 50	-	MeOH 100	-0.614	0.674
		**MeOH 50**	**-**	**DMSO 10**	**3.940**	**0.000**
		**MeOH 50**	**-**	**DMSO 50**	**3.401**	**0.002**
		**MeOH 100**	**-**	**DMSO 10**	**4.555**	**0.000**

		**MeOH 100**	**-**	**DMSO 50**	4.015	0.000
		DMSO 10	-	DMSO 50	0.539	0.680
**Catalase (CAT)**						
	Shapiro-Wilk normality test				W=0.97459	0.3542
	Bartlett test of homogeneity of variances				k^2 =5.5342	0.3542
	ANOVA				sum^2 (5)=0.8509	<0,0001
	Tukey multiple comparisons of means	NC1	-	EtOH	0.024	0.997
		NC1	-	MeOH 50	0.073	0.714
		**NC1**	**-**	**MeOH 100**	**-0.243**	**0.000**
		NC1	-	DMSO 10	0.129	0.144
		NC1	-	DMSO 50	0.122	0.186
		EtOH	-	MeOH 50	-0.050	0.927
		**EtOH**	**-**	**MeOH 100**	**0.266**	**0.000**
		EtOH	-	DMSO 10	0.106	0.332
		EtOH	-	DMSO 50	0.099	0.404
		**MeOH 50**	**-**	**MeOH 100**	**-0.316**	**0.000**
		MeOH 50	-	DMSO 10	0.056	0.886
		MeOH 50	-	DMSO 50	0.049	0.930
		**MeOH 100**	**-**	**DMSO 10**	**0.372**	**0.000**
		**MeOH 100**	**-**	**DMSO 50**	**0.365**	**0.000**
		DMSO 10	-	DMSO 50	0.007	1.000

SUB-EXPERIMENT 2						
Cellular energy allocation (CEA)						
	Shapiro-Wilk normality test				*W=0.8102*	*<0.0001*
	Levene's Test for Homogeneity of Variance (center = median)				*F=3.0614*	*0.0655*
	Kruskal-Wallis rank sum test				*χ2 (2)= 0.469*	*0.7909*
Lipid peroxidation (LPO)						
	Shapiro-Wilk normality test				*W=0.9039*	*0.0164*
	Levene's Test for Homogeneity of Variance (center = median)				*F=0.1595*	*0.8535*
	Kruskal-Wallis rank sum test				*χ2 (2)=17.173*	*0.0002*
	Dunn´s post hoc comparison	*NC2*	*-*	*DMF 50*	***-1.218***	*0.223*
		NC2	***-***	***DMF 100***	***-3,9574***	***0.000***
		DMF 50	***-***	***DMF 100***	***-2.822***	***0.007***
Catalase (CAT)						
	Shapiro-Wilk normality test				*W=0.9327*	*0.0806*
	Bartlett test of homogeneity of variances				*k^2 =22.38*	*<0.0001*
	Welch-ANOVA				*F=2.1035*	*0.1677*

S.3.3 Statistics for CEA biomarker analysis (i.e., carbohydrates (CBH), lipids, proteins, energy transport system (ETS)) of the two sub-experiments.

Table S6: Statistical analysis for energy reserves (i.e., carbohydrates, lipids, protein) and energy consumption (i.e., energy transport system) for CEA calculations of each sub-experiment (grey background= comparisons with controls; bold= indicating statistical significances).

Biomarker	Statistical Test	Comparison			Statistic	p-value
**SUB-EXPERIMENT 1**						
**Carbohydrates (CBH)**						
	Shapiro-Wilk normality test				W=0.9784	0.4339
	Bartlett test of homogeneity of variances				k^2= 15.775	0.0075
	Welch ANOVA				F=4.7241	0.0047
	Games Howell post hoc comparison	**Group1**	**-**	**Group2**		
		NC1	-	EtOH	-0.343	0.998
		NC1	-	MeOH 50	-0.274	0.999
		NC1	-	MeOH 100	1.327	0.622
		NC1	-	DMSO 10	1.696	0.426
		NC1	-	DMSO 50	0.461	0.990
		EtOH	-	MeOH 50	-0.069	1.000
		EtOH	-	MeOH 100	-1.670	0.130
		EtOH	-	DMSO 10	2.039	0.074
		EtOH	-	DMSO 50	0.804	0.702
		**MeOH 50**	**-**	**MeOH 100**	**1.601**	**0.042**
		**MeOH 50**	**-**	**DMSO 10**	**1.970**	**0.034**
		MeOH 50	-	DMSO 50	0.735	0.373
		MeOH 100	-	DMSO 10	0.369	0.992
		MeOH 100	-	DMSO 50	-0.866	0.591
		DMSO 10	-	DMSO 50	1.235	0.353
**Lipids**						
	Shapiro-Wilk normality test				W= 0.8366	<0.0001
	Levene's Test for Homogeneity of Variance (center = median)				F=2.3079	0.0587
	Kruskal-Wallis rank sum test				$\chi2$ (5)=7.3613	0.1951
**Protein**						
	Shapiro-Wilk normality test				W= 0.9352	0.006
	Levene's Test for Homogeneity of Variance (center = median)				F=0.8932	0.4932
	Kruskal-Wallis rank sum test				$\chi2$ (5)=24.286	0.0002
	Dunn´s post hoc comparison	**Group1**	**-**	**Group2**		
		NC1	-	EtOH	-0.959	0.422
		NC1	-	MeOH 50	-1.378	0.280
		**NC1**	**-**	**MeOH 100**	**-2.562**	**0.039**
		NC1	-	DMSO 10	-2.128	0.083
		**NC1**	**-**	**DMSO 50**	**-4.525**	**0.000**
		EtOH	-	MeOH 50	0.420	0.675
		EtOH	-	MeOH 100	1.603	0.204

		EtOH	-	DMSO 10	-1.169	0.331
		**EtOH**	**-**	**DMSO 50**	**-3.566**	**0.003**
		MeOH 50	-	MeOH 100	-1.184	0.331
		MeOH 50	-	DMSO 10	-0.749	0.524
		**MeOH 50**	**-**	**DMSO 50**	**-3.146**	**0.008**
		MeOH 100	-	DMSO 10	0.434	0.675
		MeOH 100	-	DMSO 50	-1.963	0.106
		DMSO 10	-	DMSO 50	2.397	0.050
**Electron Transport System (ETS)**						
	Shapiro-Wilk normality test				W=0.5043	<0.0001
	Levene's Test for Homogeneity of Variance (center = median)				F=2.3229	0.0573
	Kruskal-Wallis rank sum test				χ2 (5)=34.479	<0.0001
	Dunn´s post hoc comparison	**Group1**	**-**	**Group2**		
		NC1	-	EtOH	1.019	0.450
		NC1	-	MeOH 50	0.974	0.450
		**NC1**	**-**	**MeOH 100**	**-2.787**	**0.011**
		**NC1**	**-**	**DMSO 10**	**-2.532**	**0.019**
		**NC1**	**-**	**DMSO 50**	**-2.652**	**0.015**
		**EtOH**	**-**	**MeOH 50**	**0.045**	**0.964**
		**EtOH**	**-**	**MeOH 100**	**3.805**	**0.001**
		**EtOH**	**-**	**DMSO 10**	**-3.551**	**0.001**
		**EtOH**	**-**	**DMSO 50**	**-3.671**	**0.001**
		**MeOH 50**	**-**	**MeOH 100**	**-3.761**	**0.001**
		**MeOH 50**	**-**	**DMSO 10**	**-3.506**	**0.001**
		**MeOH 50**	**-**	**DMSO 50**	**-3.626**	**0.001**
		MeOH 100	-	DMSO 10	0.255	0.964
		MeOH 100	-	DMSO 50	0.135	0.964
		DMSO 10	-	DMSO 50	0.120	0.964

**Parameter**	**Statistical Test**	**Comparison**			**Statistic**	**p-value**
**SUB-EXPERIMENT 2**						
**Carbohydrates (CBH)**						
	Shapiro-Wilk normality test				W=0.9713	0.6374
	Bartlett test of homogeneity of variances				k^2= 1.7044	0.4265
	Welch ANOVA				F=0.6365	0.5423
**Lipids**						
	Shapiro-Wilk normality test				W= 0.7257	<0.0001
	Levene's Test for Homogeneity of Variance (center = median)				F=0.3415	0.7141
	Kruskal-Wallis rank sum test				χ2 (2)=2.6435	0.2667
**Protein**						
	Shapiro-Wilk normality test				W= 0.9297	0.0681
	Bartlett test of homogeneity of variances				k^2=5.1468	0.0763
	ANOVA				sum^2 (2)=21.12	0.0032
	Dunn´s post hoc comparison	**Group1**	**-**	**Group2**		

Internal

		NC1	***-***	***DMF 50***	***-1.979***	***0.005***
		NC1	***-***	***DMF 100***	***-1.752***	***0.013***
		DMF 50	*-*	*DMF 100*	*0.227*	*0.915*
Electron Transport System (ETS)						
	Shapiro-Wilk normality test				*W=0.7102*	*<0.0001*
	Levene's Test for Homogeneity of Variance (center = median)				*F=1.7421*	*0.1966*
	Kruskal-Wallis rank sum test				*χ2 (5)=9.6508*	*0.008*
	Dunn´s post hoc comparison	***Group1***	***-***	***Group2***		
		NC1	***-***	***DMF 50***	***-2.851***	***0.013***
		NC1	***-***	***DMF 100***	***-2.494***	***0.019***
		DMF 50	*-*	*DMF 100*	*0.356*	*0.722*

S4 – References:

1. Borneman, E. Introduction to the husbandry of corals in aquariums: A review. *Public Aquarium Husbandry Series* **2008**, *2*, 3-14.
2. Fosså, S.A.; Nilsen, A.J. *Das Korallenriff-Aquarium*; Natur und Tier Verlag GmbH: Münster, **2013**.
3. TropicMarin. Supply System. Available online: https://www.tropic-marin.com/versorgungssysteme?lang=en (accessed on 03/2023)
